# Development of the nervous system in *Platynereis dumerilii* (Nereididae, Annelida)

**DOI:** 10.1186/s12983-017-0211-3

**Published:** 2017-05-25

**Authors:** Viktor V. Starunov, Elena E. Voronezhskaya, Leonid P. Nezlin

**Affiliations:** 1 Department of Invertebrate Zoology, St-Petersburg State University, St-Petersburg, 199034 Russia; 2 Zoological Institute Rus, Acad. Sci, St-Petersburg, 199034 Russia; 3 Institute of Developmental Biology, Rus. Acad. Sci, Moscow, 119991 Russia

**Keywords:** *Platynereis dumerilii*, Annelida, Trochophora, Neuronal development, Serotonin, FMRFamide, Tubulin, Confocal microscopy

## Abstract

**Background:**

The structure and development of the nervous system in Lophotrochozoa has long been recognized as one of the most important subjects for phylogenetic and evolutionary discussion. Many recent papers have presented comprehensive data on the structure and development of catecholaminergic, serotonergic and FMRFamidergic parts of the nervous system. However, relatively few papers contain detailed descriptions of the nervous system in Annelida, one of the largest taxa of Lophotrochozoa. The polychaete species *Platynereis dumerilii* has recently become one of the ﻿more popular model animals in evolutionary and developmental biology. The goal of the present study was to provide a detailed description of its neuronal development. The data obtained will contribute to a better understanding of the basic features of neuronal development in polychaetes.

**Results:**

We have studied the development of the nervous system in *P. dumerilii* utilizing histo- and immunochemical labelling of catecholamines, serotonin, FMRFamide related peptides, and acetylated tubulin. The first neuron differentiates at the posterior extremity of the protrochophore, reacts to the antibodies against both serotonin and FMRFamide. Then its fibres run forwards along the ventral side. Soon, more neurons appear at the apical extreme, and their basal neurites form the basel structure of the developing brain (cerebral neuropil and circumesophageal connectives). Initial development of the nervous system starts in two rudiments: anterior and posterior. At the nectochaete stage, segmental ganglia start to differentiate in the anterior-to-posterior direction, and the first structures of the stomatogastric and peripheral nervous system appear. All connectives including the unpaired ventral cord develop from initially paired nerves.

**Conclusions:**

We present a detailed description of *Platynereis dumerilii* neuronal development based on anti-acetylated tubulin, serotonin, and FMRFamide-like immunostaining as well as catecholamine histofluorescence. The development of the nervous system starts from peripheral pioneer neurons at both the posterior and anterior poles of the larva, and their neurites form a scaffold upon which the adult central nervous system develops. The anterior-to-posterior mode of the ventral ganglia development challenges the primary heteronomy concept. Comparison with the development of Mollusca reveals substantial similarities with early neuronal development in larval Solenogastres.

**Electronic supplementary material:**

The online version of this article (doi:10.1186/s12983-017-0211-3) contains supplementary material, which is available to authorized users.

## Background

During the past decades, our understanding of bilaterian ontogeny, history and taxonomy has changed considerably. Based on a set of comparative morphological and genetic traits, three major groups of bilaterally symmetrical animals are currently identified: Deuterostomia (chordates, hemichordates and echinoderms), Ecdysozoa (arthropods, nematodes and several smaller phyla) and Lophotrochozoa (molluscs, annelids, nemerteans, sipunculids, phoronids and bryozoans) [1–6]. Popular animal models mainly belong to ecdysozoans and deuterostomes (e.g. *Caenorhabditis*, *Drosophila*, mouse), whereas lophotrochozoans are still largely ignored. Detailed morphological and developmental studies of model lophotrochozoans are required to address questions about the evolution in the three main bilaterian clades.

Recently, the annelid *Platynereis dumerilii* (Audouin & Milne Edwards, 1834) has been established as a promising animal model for developmental, evolutionary and ecological research [6–11]. This marine animal is well suited as a model system for several reasons.

1. *Platynereis* exhibits a canonical biphasic pelagobenthic life cycle involving a pelagic free swimming primary larva, and benthic adults. Egg cleavage follows the typical spiral model, and the canonical course of cleavages produces a typical trochophore larva [10]. Besides, *Platynereis* exhibits modes of development and body plans that are considered ancestral in many respects. This is also reflected in the level of genes, making this animal ideally suited for developmental comparative studies [6].
2. This animal will breed in a laboratory culture without access to the sea, and produce offspring continuou﻿sly throughout the year. With 2000–3000 synchronously developing eggs, a single clutch can be split up into numerous samples for serial or parallel observations [10].
3. Both larval and juvenile stages are transparent and well suited for light microscopy including confocal laser scanning microscopy (CLSM) in combination with whole mount immunohistochemistry [11, 12].
4. *Platynereis* has a long story of classical embryological investigations, and many aspects of its development have been described in great detail [9, 12–15]. Thus, the study of this animal will provide comparative data that may contribute to better understanding of the basic plan of annelid development and evolution.

Our understanding of neuronal development in Lophotrochozoa has increased greatly over the last decades due to numerous comparative morphological studies, which used fluorescent histo- and immunocytochemical labelling in combination with confocal microscopy. Identification of catecholaminergic, serotonergic and FMRFamide-like immunoreactive nerve elements have been shown to be reliable neuron-specific markers that permit the detection of neurons, fibre tracts and isolated fibres in whole mount preparations [16, 17], while co-staining with the antibody against acetylated α-tubulin reveals gross morphology of the nervous system and ciliated structures [18]. Based on these techniques, detailed descriptions of neuronal development and larval nervous systems in representative species of molluscs [19–24], phoronids [25–28], bryozoans [29–31], brachiopods [32], echiurids [33, 34], and sipunculids [35] have been published.

Modern evolutionary studies have revealed substantial similarities in the molecular architecture of the nervous system in annelids, arthropods and vertebrates, suggesting that this architecture was present in the last common bilaterian ancestor and supporting a common origin of nervous system centralization in Bilateria [36–40]. Thus, the study of neuronal development in polychaetes may shed light on CNS evolution in Bilateria.

However, only few papers describe the development of the nervous system in polychaetes and the data is sometimes contradictory. Hay-Schmidt [41] describes in *Polygordius lacteus* (Polygordiidae) the development of a highly centralized serotonin- and FMRFamide-like immunoreactive (lir) nervous system, with the first neurons appearing within the developing central ganglia and nerve cords. Later, the study of neuronal development in planctotrophic larvae of errant *Phyllodoce maculata* (Phyllodocidae) [42] and sedentary *Pomatoceros lamarckii* (Serpulidae) [43] revealed that the first neurons to appearing in early trochophores was at the periphery. In both species, the first serotonergic cell appeared at the posterior end and the first FMRFa-lir cell at the anterior end of the larva. In larval *Sabellaria alveolata* (Sabellaridae) [44] the first serotonergic fibres were detected at later stages at the tip of each chaetal sac. Recently, Fischer and co-authors [11] provided an overview of neuronal development in lecitotrophic larvae of *Platynereis dumerilii* (Nereididae) until the late nectochaete stage based on anti-serotonin and anti-tubulin immunostaining. As to the catecholaminergic nervous system, its development remains completely unknown throughout the whole phylum Annelida, whereas several papers have reported distribution of catecholamine-synthesizing neurons in adult polychaetes [45–48] and medicinal leeches [49].

The goal of our research was to study in detail the neuronal development in *P. dumerilii* from a comparative and developmental perspective using immunocytochemical labelling with commercial antibodies against acetylated α-tubulin, FMRFamide and serotonin, as well as the histochemical staining of the catecholamines. Below, we have provided detailed descriptions of the development of anti-5-HT and anti-FMRFamide-like immunostainings in the nervous system of *P﻿. dumerilii* from hatching until the formation of the juvenile worm with 10–11 segments. Catecholamine-containing structures were studied until the late nectochaete stage. Where possible, we have used the﻿ neuroanatomical terms suggested by Richter and co-authors [50]. Since the development of serotonergic elements of the nervous system until the mid-nectochaete stage was described earlier [11], we report it only in brief, focusing on newly observed details. Taking into account the low specificity of the FMRFamide antibody, which cross-reacts with many neuropeptides that have a RFamide terminus, we compared our results only with similar data from other trochozoan species. Detailed descriptions of the development of serotonergic, FMRFamidergic and catecholaminergic nervous elements in *Platynereis* will contribute to better understanding of the basic features of neuronal development and shed light on phylogenetic relationships within Trochozoa.

## Results

### Staging of development

Normal development of *P.dumerilii* has been described in great detail [11]. Since the pace of development is highly temperature dependent, we determined developmental stages according to the set of morphological and behavioural characters as described in [11]. Precise time points of fixation (in hours or days post-fertilisation) for each preparation are indicated in the figure legends.

### FMRFa-like immunoreactivity (Figs. 1, 2 and 3)

**Fig. 1:**
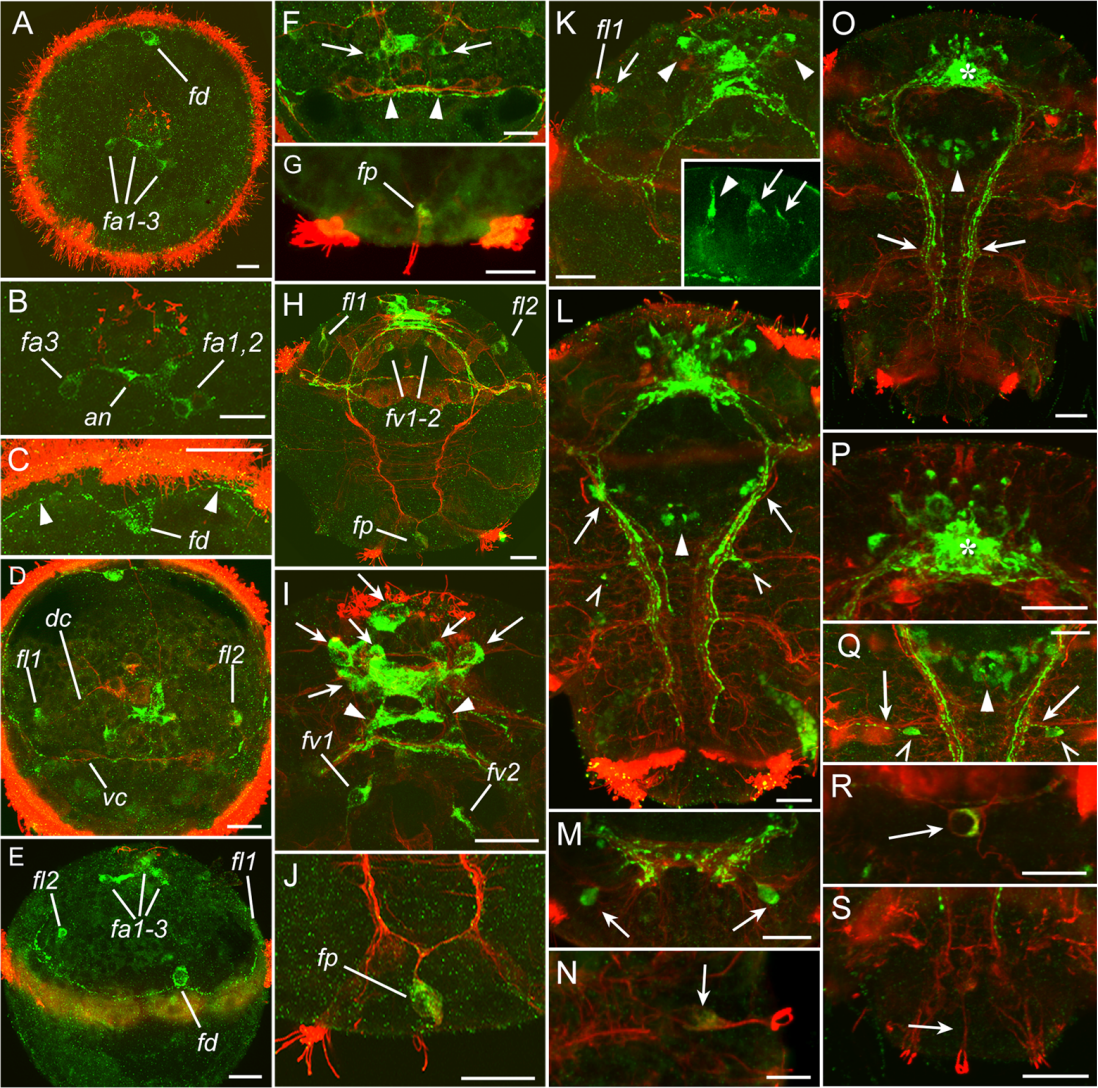
Development of FMRFamide-like (*green*) and acetylated tubulin-like (*red*) immunoreactivity in *P. dumerilii*. Early trochophore (**a-c**), mid-trochophore (**d-e**), late trochophore (**f-g**), early metatrochophore (**h-j**), mid-metatrochophore (**k**), late metatrochophore (**l-n**), early nectochaete (**o-s**). **a:** 24 hpf, apical view. The first immunopositive cells are three apical neurons (*fa1–3*) and a solitary dorsal neuron (*fd*). **b:** 24 hpf, apical neurons at high magnification. The neurons *fa1,2* are located to the left and *fa3* to the right of apical neuropil *(an)*. **c**: 24 hpf, high magnification of the dorsal neuron *fd*. Two neurites (arrowheads) extend from it in opposite directions beneath the prototroch. **d:** 28 hpf, apical view showing the cells *fl1* and *fl2*. *dc* and *vc* – dorsal and ventral cerebral commissures. **e:** 32 hpf, dorsal view showing the cells *fa1–3*, *fd*, and *fl2*. **f:** 36 hpf, apical view showing neurons in the apical organ (*arrows*) and ventral cerebral commissure (*arrowheads*). **g:** 36 hpf, ventral view of the solitary posterior cell (*fp*) with two apical cilia. **h:** 45 hpf, ventral view showing two ventral pretrochal cells (*fv1,2*) and lateral cells (*fl1*,*2*). **i:** 45 hpf, ventral view on the apical region showing apical neurons (*arrows*), cerebral ganglion (*arrowheads*) and ventral pretrochal cells (*fv1, fv2*). **j:** 45 hpf, ventral view on the posterior cell (*fp*). **k:** 50 hpf, ventral view of the pretrochal region showing FMRFa immunoreactivity in the apical region (*arrowheads*) and two cells (*arrow*) close to the cell *fl1*. *Inset*: High magnification of the two cells (*arrows*) close to the cell *fl1* (*arrowhead*)*.* **l:** 56 hpf, ventral view. Two groups of neurons associated with the ventral nerve cord (*arrows*), ventral neurons behind the stomatogastric nerve ring (*arrowhead*), and two neurons in the first chaetigerous segment (*open arrowheads*) are seen. **m:** 56 hpf, the neurons of the first chaetigerous segment (*arrows*). **n:** 56 hpf, posterior cell *fp* (*arrow*). **o:** 72 hpf, ventral view showing the brain (*asterisk*), neurons behind the stomatogastric nerve ring (*arrowhead*), and paired ventral nerve cord (*arrows*). **p:** 72 hpf, brain region with dense neuropil (*asterisk*). **q:** 72 hpf, anterior part of the ventral nerve cord showing neurons behind the stomatogastric nerve ring (*arrowhead*), and two neurons in the first chaetigerous segment (*open arrowheads*) sending fibers into the ventral cord and segmental nerves (*arrows*). **r:** 72 hpf, dorsal cell *fd* (*arrow*). **s:** 72 hpf, posterior cell *fp* (*arrow*) with no FMRFa immunoreactivity. Scale bars = 20 μm

The first FMRFa-like immunoreactive (FMRFa-lir) cells were detected in early trochophores shortly after hatching. Three neurons were located almost ventrally to the apical extreme (FMRFa-lir apical, *fa1–3*, Fig. 1a,b) and projected their basal neurites into a compact neuropil. In addition, one bipolar cell was located dorsally below the prototroch (FMRFa-lir dorsal, *fd*, Fig. 1a,c). Two neurites extended from the soma and ran in both directions beneath the prototrochal cells forming part of the trochal neurite bundle (prototroch nerve).

**Fig. 2:**
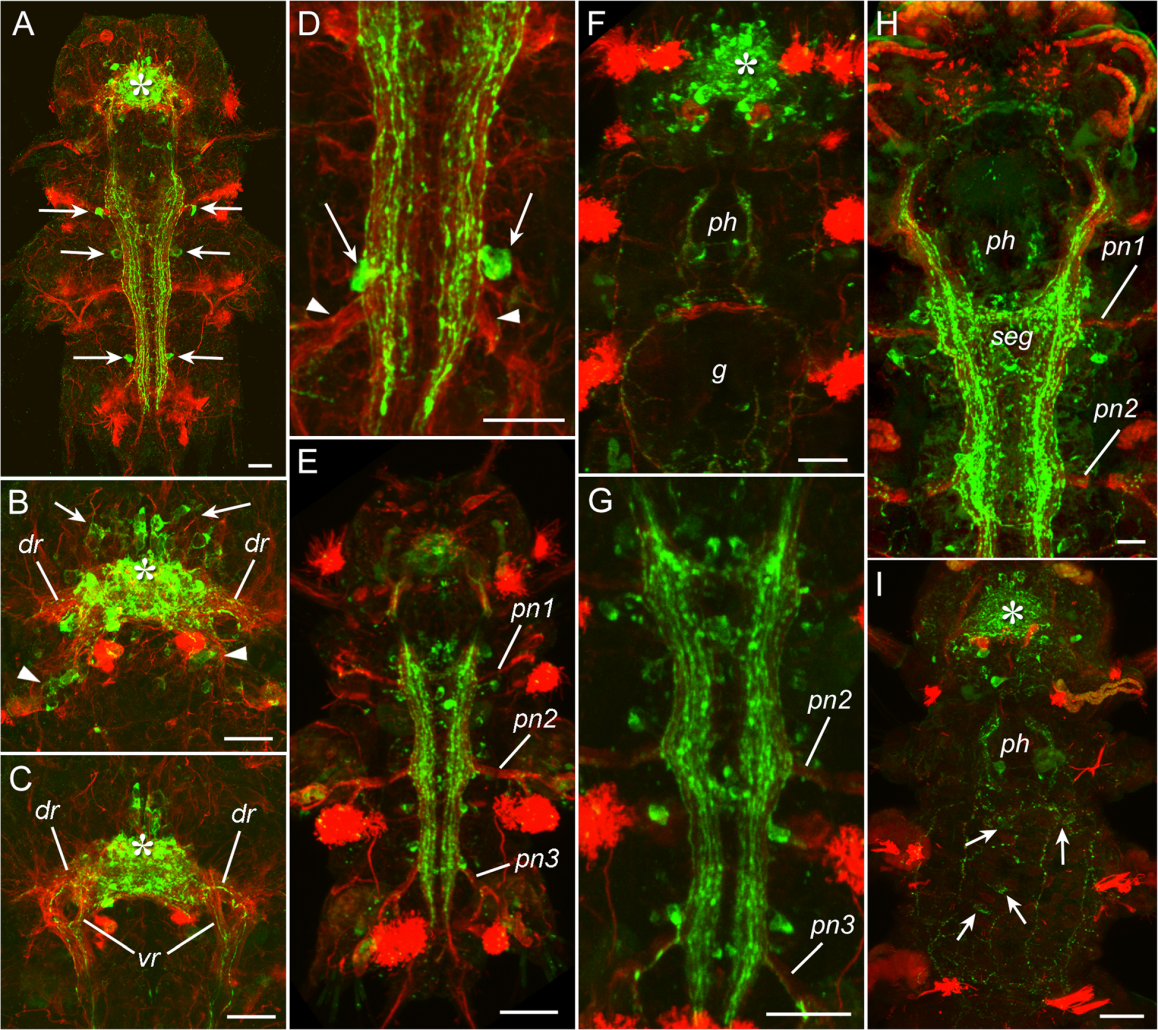
Development of FMRFamide-like (green) and acetylated tubulin-like (*red*) immunoreactivity in *P. dumerilii*. Mid-nectochaete (**a-d**), late nectochaete (**e-g**), three-segmented errant juvenile worm (**h-i**). **a:** 3,5 dpf, ventral view showing the brain (*asterisk*) and the paired ventral cord. *Arrows* indicate the pairs of neurons in the larval segments 1–3. **b:** dorsal neuropil with FMRFa-lir neurons anterior (*arrows*) and posterior (*arrowheads*) to it; *dr* – dorsal roots of circumoesophageal connectives. **c:** Ventral neuropil; *dr* – dorsal roots of circumoesophageal connectives, *vr* – ventral roots of circumoesophageal connectives. **d:** Posterior part of the ventral cord with two neurons (*arrows*) in the third chaetigerous segment near the roots of parapodial nerves (*arrowheads*). **e:** 7 dpf, ventral view showing FMRF-lir structures in the body. *Pn1–3*– parapodial nerves of corresponding chaetigerous segments. **f:** dorsal view on the anterior body part showing brain neuropil (*asterisk*), innervation of the pharynx (*ph*) and gut (*g*). **g:** ventral nerve cords with adjacent FMRF-lir neurons. **h:** 16 dpf, FMRF-lir innervation of the ventral body part showing the subesophageal ganglion (*seg*) and pharynx (*ph*). **i:** FMRF-lir innervation of the dorsal body part showing brain neuropil (*asterisk*) with adjacent neurons, pharynx innervation (*ph*), and dorsal plexus with peripheral neurons (*arrows*). Scale bars = 20 μm

**Fig. 3:**
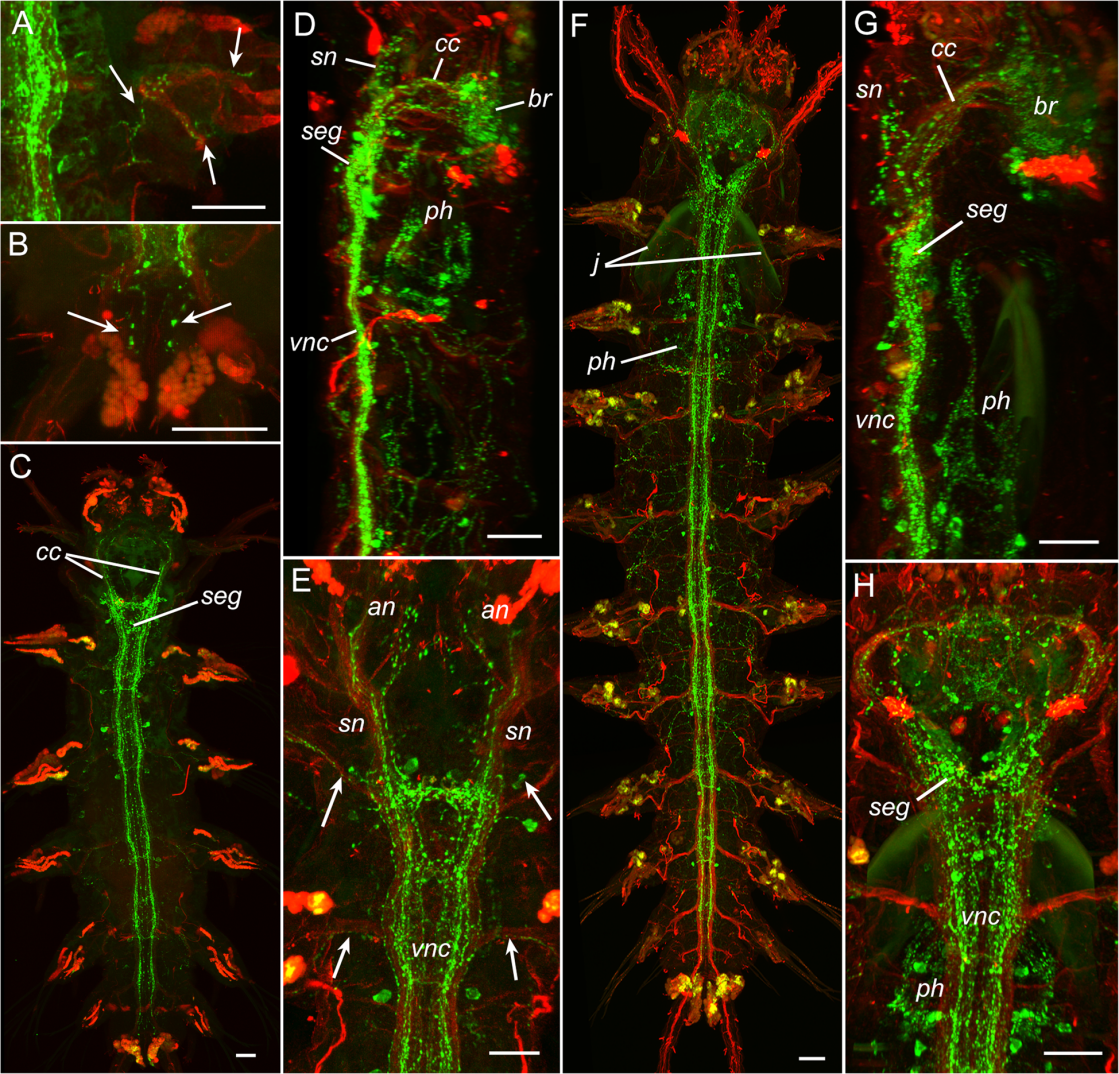
Development of FMRFamide-like (*green*) and acetylated tubulin-like (*red*) immunoreactivity in *P. dumerilii.* Three-segmented errant juvenile worm (**a-b**), cephalic metamorphosis (**c-e**), small atokous worm (**f-h**). **a:** 16 dpf, FMRF-lir innervation of the parapodium (*arrows*). **b:** 16 dpf, FMRF-lir innervation of the pygidium (*arrows*). **c-e:** 6-segmented juvenile, 20 dpf; ventral view showing FMRFa-lir innervation of the body (**c**); left lateral (**d**) and ventral (**e**) view on the anterior body part showing FMRFa-lir immunoreactivity in the brain (*br*), circumesophageal connectives (*cc*), stomatogastric nerves (*sn*), subesophageal ganglion (*seg*), pharynx (*ph*), antennal nerves (*an*), segmental nerves (*arrows*) and ventral nerve cord (*vnc*). See additional movie [Additional file 1] for 3D rotatable reconstruction. **f-h:** 10-segmented juvenile, 25 dpf, ventral view showing FMRFa-lir innervation of the body (**f**); left lateral (**g**) and ventral (**h**) view on the anterior body part showing FMRFa-lir immunoreactivity in the brain (*br*), circumesophageal connectives (*cc*), stomatogastric nerves (*sn*), subesophageal ganglion (*seg*), pharynx (*ph*), and ventral nerve cord (*vnc*); *j* - jaws. See additional movie [Additional file 2] for 3D rotatable reconstruction. Scale bars = 20 μm

At the beginning of the mid-trochophore stage, two more cells that sent neurites into the trochal neurite bundle appeared in the lateral portions of the episphere (left and right FMRFa-lir lateral, *fl1–2*, Fig. 1d). Immunopositive neurites were detected in the apical neuropil, prototroch nerve and in the developing ventral cerebral commissure (*vc*, Fig. 1d). By 32 hpf, the set of FMRFa-lir cells remained unchanged (Fig. 1e): three asymmetrically located cells (*fa1–3*) in the apical region, the dorsal cell (*fd*) and the pair of lateral cells (*fl1* and *fl2*). No immunoreactivity was yet detected in the hyposphere.

At the late trochophore stage, the number of immunopositive cells in the apical organ increased to six and the first immunopositive cell bodies appeared in the region of the developing brain (ventral cerebral commissure) (*arrowheads*, Fig. 1f). At this time, faint FMRFa-like immunoreactivity was detected in the soma of the solitary cell located at the posterior extreme of the larva inside the telotroch ring (FMRFa-lir posterior, *fp,* Fig. 1g). The cell had an apical dendrite with surface cilia.

At the early metatrochophore stage, the same six cells in the apical organ, one pair of cells in the developing brain (cerebral ganglion), and the cell *fp* were detected (Fig. 1h,i). A pair of ciliated FMRFa-lir cells appeared at the ventral surface of the episphere (*fv1–2,* Fig. 1h), and their fibres projected into the prototrochal neurite bundle. Based on their position and the presence of the dendrite and projections to the prototrochal nerve, these cells represent the photoreceptor cells of the eyespot. In the hyposphere, the *fp* cell was still the only FMRFa-lir structure (Fig. 1h,j). Relative to other immunopositive structures, the intensity of staining in this cell was always very weak in the soma and almost undetectable in the basal fibres. At the mid-metatrochophore stage, more cells appeared in the apical region, and a pair of cells appeared near each of the cells *fl1* and *fl2* (Fig. 1k).

At the late metatrochophore stage, more FMRFa-lir neurons appeared in the apical region, and the neuropil expanded (Fig. 1l). Immunopositive cells appeared ventrally to the posterior part of the stomatogastric nerve ring (Fig. 1l, *arrowhead*), and two groups of neurons appeared in association with the ventral nerve cords dorsal to the trochal neurite bundle (Fig. 1l, *arrows*). Immunoreactive fibres appeared in the posttrochal parts of the paired ventral cord. Two symmetrical FMRFa-lir neurons appeared in the first chaetigerous segment (Fig. 1l, *open arrows*; M, *arrows*). They were located ventro-lateral to the cords and sent neurites into them. At this time, immunoreactivity in the posterior *fp* cell became very weak and soon disappeared (Fig. 1n).

At the early nectochaete stage, the adult CNS further developed, and more immunopositive elements were added to the paired ventral cord and the brain (Fig. 1o). The brain neuropil increased in size and became denser (Fig. 1p, asterisk). The number of immunopositive neurites in the ventral cord also increased, though these fibres did not reach the rear of the body. Fibres of FMRFa-lir neurons in the first chaetigerous segment projected into the ventral cord and segmental nerves (Fig. 1q, *arrows*). At this time, immunoreactivity in the dorsal *fd* cell started to fade (Fig. 1r, *arrow*). No immunoreactivity was detected in the posterior *fp* cell though the cell body could be visualized after anti-tubulin immunostaining being the only ciliated cell inside the telotroch ring (Fig. 1s, *arrow*).

At the mid-nectochaete stage, more FMRFa-lir neurons appeared in the head region. More neurites were added to the paired ventral cord and projected into its posterior end. Two pairs of immunopositive cells appeared in the second and third chaetigerous segments (Fig. 2a, *arrows*). In the dorsal part of the brain, cell bodies were detected anterior and posterior to the neuropil (Fig. 2b). In the ventral part, although no neurons were added the central neuropil grew in size (Fig. 2c). Immunopositive fibres were detected in the dorsal and ventral roots of the circumoesophageal connectives (Fig. 2b,c, *dr*, *vr*). The cells in the first and third segment were located near the roots of parapodial nerves (Fig. 2d). No FMRFa-lir innervation was as yet detected in the tentacular cirri, anal cirri and antennal stubs that were forming at this stage.

Starting from the late nectochaete stage, the number of immunoreactive neurons and neurites in the adult CNS, including the brain, dorsal neuropil and ventral cord started to increase (Fig. 2e-g), and FMRFa-lir innervation of the pharynx (*ph*) and gut (*g*) was first detected (Fig. 2f). In three-segmented errant juvenile worms (Fig. 2h,i), a dense network of immunopositive fibres was detected in the ventral cord (*vc*) and subesophageal ganglion (*ceg*), and a plexus of FMRFa-lir neurones and neurites appeared in the dorsal portion of the body (Fig. 2i, *arrows*). At this time, FMRFa-lir neurites were first detected to innervate the parapodia (Fig. 3a, *arrows*) and the pygidium (Fig. 3b, *arrows*). At the periphery, the number of neurons and neurites continued to increase.

At the stage of cephalic metamorphosis, immunoreactive cells and fibres were detected in the central and peripheral nervous system (Fig. 3c-e, Additional file 1). In the anterior region, FMRFa-lir neurons and neurites were present in almost all parts of the CNS including the brain (*br*), ventral nerve cord (*vnc*), circumesophageal connectives (*cc*), subesophageal ganglion (*ceg*), antennal (*an*), stomatogastric (*sn*) and segmental nerves (*arrows*). FMRFa-lir innervation was also found in the digestive system (pharynx and gut) and body wall (Fig. 3d,e).

At the stage of the small atokous worm, FMRFa-lir neurons and neurites were present in the central and peripheral nervous system, similar to the previous stage although the number of cells and neurites had increased (Fig. 3f-h, Additional file 2). A peripheral plexus of immunoreactive neurites and scattered neurons were detected along the length of the body.

### Serotonin-like immunoreactivity (Figs. 4 and 5)

**Fig. 4:**
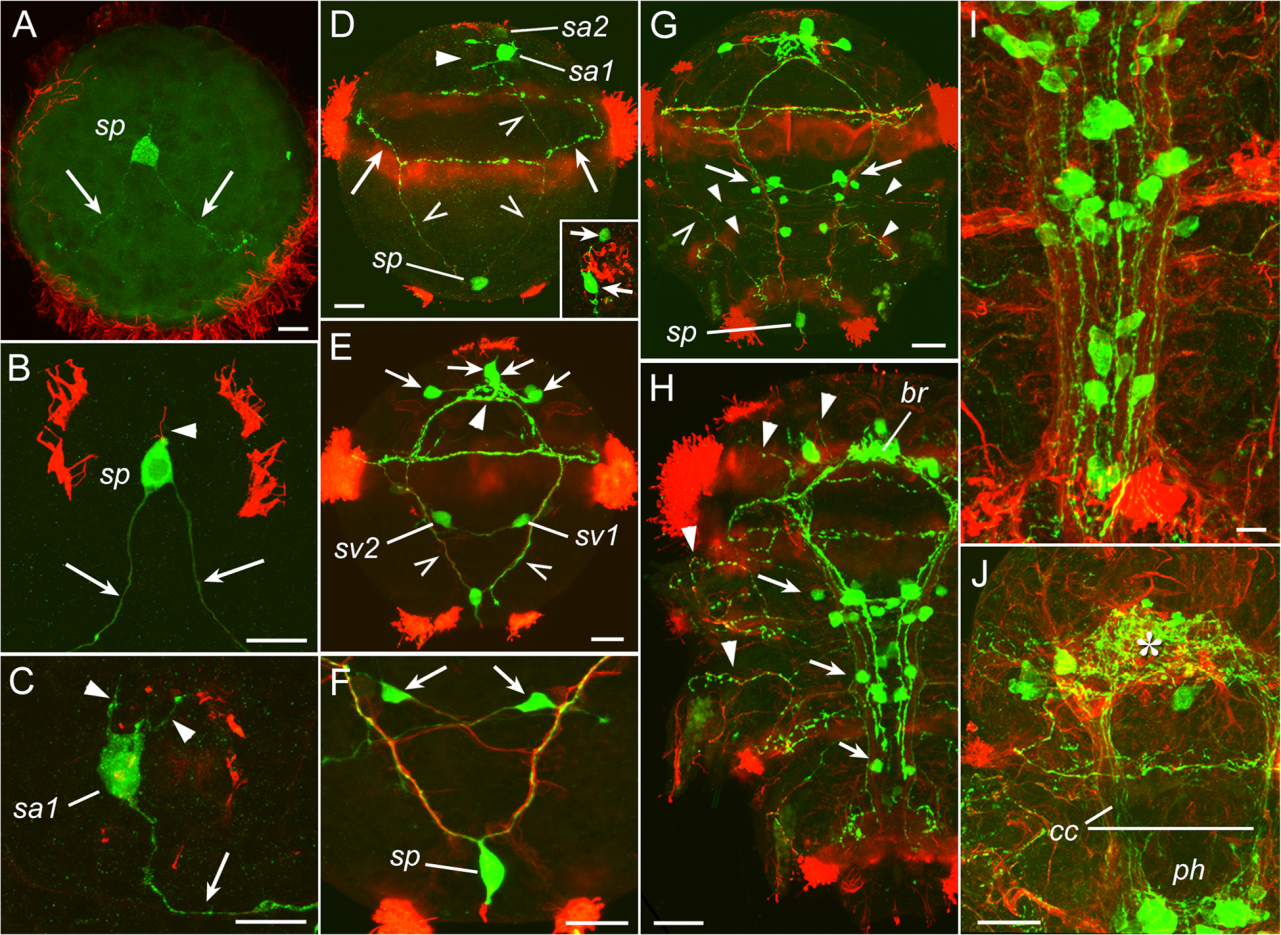
Development of 5-HT-like (*green*) and acetylated tubulin-like (*red*) immunoreactivity in *P. dumerilii*. Protrochophore (**a**), early trochophore (**b,c**), mid-trochophore (**d**), early metatrochophore (**e,f**), mid-metatrochophore (**g**), early nectochaete (**h**), late nectochaete (**i,j**). **a:** 17 hpf, caudal view on the first posterior 5-HT-lir cell (*sp*) with two basal fibers (*arrows*). **b:** 20 hpf, the first posterior 5-HT-lir cell with two basal fibers (*arrows*) and two apical cilia (*arrowhead*) in the centre of the open-circuited telotroch ring. **c:** 20 hpf, apical view on the first apical 5-HT-lir cell (*sa1*) with two short fibers (*arrowheads*) running at the base of the horseshoe-shaped apical ciliary tuft, and the long fibre running towards the prototroch (*arrow*). **d:** 24 hpf, ventral view showing the cells *sp.*, *sa1* and *sa2*, and 5-HT-lir fibres labelling the apical neuropil (*arrowhead*), prototroch nerve (*arrows*) and paired ventral cords (*open arrows*). Inset: 28 hpf, apical view showing the cells *sa1* and *sa2* (*arrows*). **e:** 44 hpf, ventral view showing four 5-HT-lir cells *sa1–4* (*arrows*) around the apical neuropil (*arrowhead*) and two ventral cells (*sv1* and *sv2*) near the ventral cords (*open arrows*). **f:** 44 hpf, high magnification of the posttrochal body part showing the posterior cell *sp.* and two ventral cells *sv1* and *sv2* (*arrows*) with their fibres running into the ventral cords crosswise. **g:** 46 hpf, ventral view showing 5-HT-lir cells associated with the ventral cords (*arrows*), transversal nerves (*arrowheads*) and the right dorsolateral longitudinal nerve (*open arrow*). **h:** 68 hpf, ventral view showing 5-HT-lir neurons in the developing brain (*br*), ventral nerve cord (*arrows*), and neurites in the head and segmental nerves (*arrowheads*). **i**: 3.5 dpf, ventral view showing 5-HT-lir neurons and neurites in the central and peripheral nervous system; *asterisk* - cerebral ganglion; *arrows* - ganglia of larval segments. **j**: 3.5 dpf, ventral view on the head region showing 5-HT-lir neuropil in the brain (*asterisk*) and innervation of the pharynx (*ph*). *cc* – circumoesophageal connectives. Scale bars = 20 μm

The first 5-HT-lir neuron to appear was the posterior 5-HT-lir cell (serotonergic posterior, *sp*), detected at the protrochophore stage before hatching (17 hpf) (Fig. 4a). Its body was triangular or claviform, a short apical dendrite extended to the surface, and two basal fibres ran towards the prototroch along the ventral side of the larva. At the time of hatching (20 hpf), two cilia appeared on the apical dendrite of the *sp* cell (Fig. 4b, *arrowhead*), and its basal fibres reached the prototroch where each fibre bifurcated and ran under the prototroch in both directions. At this time, the first apical 5-HT-lir сell (serotonergic apical, *sa1*) appeared below the right part of the horseshoe-shaped apical tuft of cilia (Fig. 4c). The cell had two short fibres running underneath the apical cilia (*arrowheads*), and a fibre directing towards the prototroch along the ventral side of the body (*arrow*). Soon after hatching (24 hpf), basal fibres of the two 5-HT-lir cells labelled the future paired ventral nerve cord and the prototroch nerve (Fig. 4d, *arrows*). At the mid-trochophore stage, another 5-HT-lir cell (*sa2*) appeared at the apical extreme, dorsally to the *sa1* cell (Fig. 4d). By the late trochophore stage, two more neurons appeared in the apical hemisphere (Fig. 4e, *arrows*) and the basal neurites of all the cells projected into the developing apical neuropil.

**Fig. 5:**
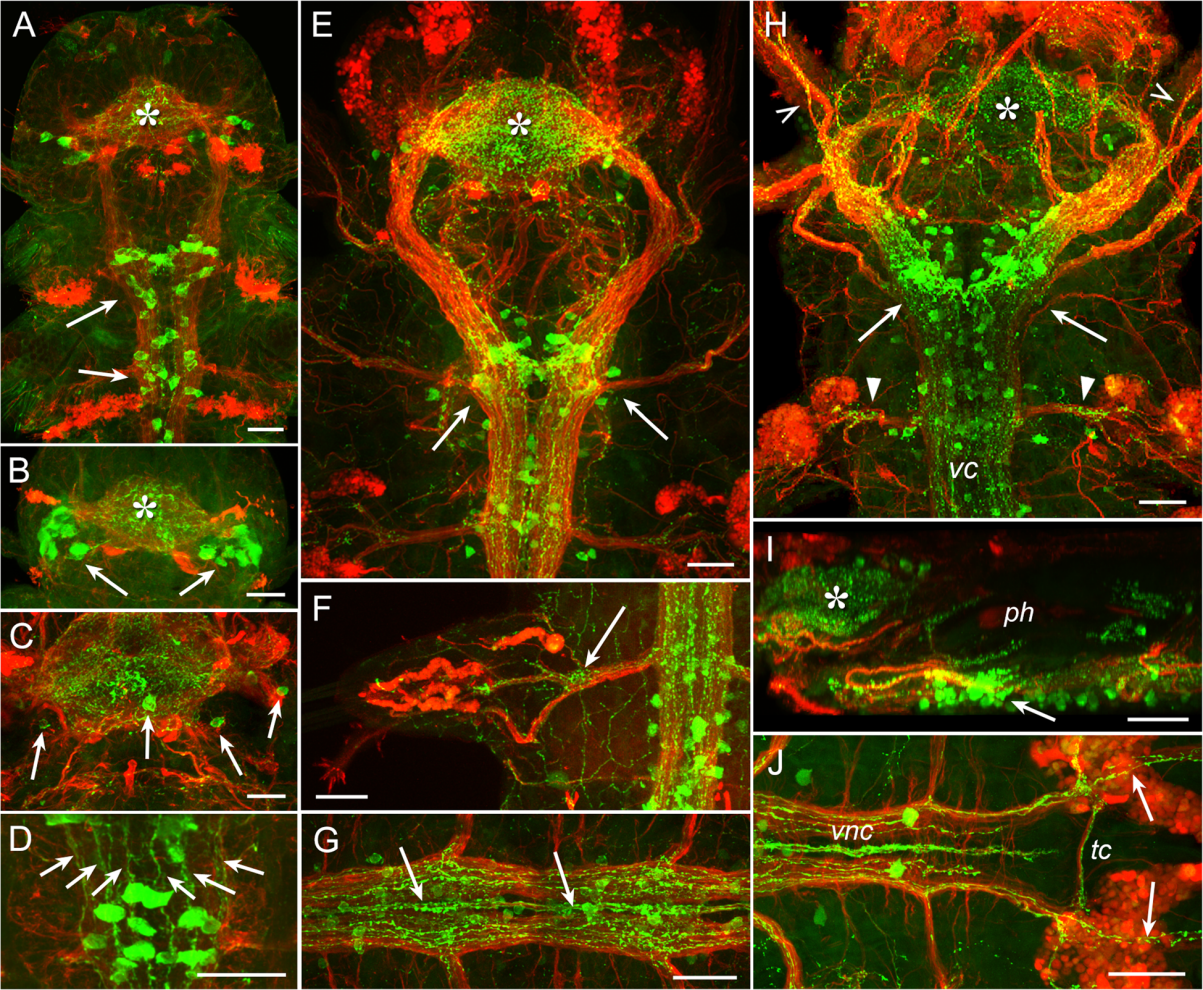
Development of 5-HT-like (*green*) and acetylated tubulin-like (*red*) immunoreactivity in *P. dumerilii*. 3-segmented larva (**a-d**), and juvenile worm (**e-j**). **a:**7 dpf, showing 5-HT-lir neurons in the cerebral ganglion (*asterisk*) and ventral cord (*arrows*). **b:** 7 dpf, dorsal part of the cerebral ganglion showing the neuropil (*asterisk*) and dorsolateral neurons (*arrows*). **c:** 7 dpf, dorsal part of the head showing apical neurons *sa1-sa4* (*arrows*). **d:** 7 dpf, 5-HT-lir neurites in the ventral cord group into six longitudinal bundles (*arrows*). **e:** 5-segmented juvenile,19 dpf, ventral view on the head region showing the brain (*asterisk*) and forming subesophageal ganglion (*arrows*). **f:** 7-segmented juvenile, 20 dpf, ventral view on the parapodium showing the parapodial ganglion (*arrow*). **g:** 7-segmented juvenile, 20 dpf, ventral nerve cord with a medial unpaired bundle of 5-HT-lir neurites (*arrows*). **h:** 10-segmented juvenile, 25 dpf, ventral view on the head region showing 5-HT-lir structures in the brain (*asterisk*), subesophageal ganglion (*arrows*), ventral cord (*vc*), tentacular nerves (*open arrows*), and parapodial nerves (*arrowheads*). **i:** 10-segmented juvenile, 25 dpf, lateral view on the head region showing 5-HT-lir structures in the brain (*asterisk*), subesophageal ganglion (*arrow*), and pharynx (*ph*). See additional movie [Additional file 3] for 3D rotatable reconstruction. **j:** 10-segmented juvenile, 25 dpf, ventral view on the caudal body end showing 5-HT-lir structures in the ventral cord (*vc*) and anal cirri (*arrows*); *tc* – terminal commissure. Scale bars = 20 μm

At the early metatrochophore stage, the first pair of 5-HT-lir neurons appeared medially to the developing paired ventral cord at the level of the first chaetigerous segment (Fig. 4e). The cells were bipolar and connected to the cord crosswise. Each cell sent a neurite into the ipsi- and contralateral part of the cord (Fig. 4f). Several hours later, more 5-HT-lir cells appeared close to the ventral cord and two pairs of transverse nerves were detected at the level of the second and third chaetigerous segments (Fig. 4g). A pair of 5-HT-lir dorsolateral longitudinal nerves extended from the prototroch backward along both dorsolateral sides of the larva (Fig. 4g, *open arrow*).

Later, the number of cells and neurites in the ventral cord started to increase and, by the early nectochaete stage, up to 24 pairs of 5-HT-lir neurons were detected along the cord (Fig. 4h). Many 5-HT-lir neurites were detected in the nerves innervating the developing palps, anal cirri and frontal head region (Fig. 4h, arrowheads). By the late nectochaete stage, the number of immunopositive neurons in the brain and the ventral cord (ganglia of chaetigerous segments) had again increased (Fig. 4i). Immunopositive neuropil’s developed in the brain and in the pharyngeal region, and numerous peripheral neurites could be seen all over the body (Fig. 4j).

In three-segmented juvenile worms, 5-HT-lir cells were detected in the brain and ventral cord (Fig. 5a). Cerebral neurons were mostly located in the dorso-lateral parts of the brain (Fig. 5b), and weakly fluorescent perikarya of the apical cells *sa1-sa4* were still visible dorsally to the cerebral ganglion (Fig. 5c). Immunoreactive fibres in the ventral cord were arranged into six paired longitudinal bundles (Fig. 5d).

In 5- to 7-segmented juveniles, the ganglia of the first and second chaetigerous segment started to merge into the subesophageal ganglion (Fig. 5e, *arrows*). The parapodia of the first chaetigerous segment including the parapodial nerves transformed into the the third pair of tentacular cirri that develops from this segment, and other segmental nerves of this segment reduced. At this time, 5-HT-lir neurites appeared in the developing parapodial ganglia (Fig. 5f, *arrow*). The number of 5-HT-lir neurites in the ventral cord gradually increased, and a medial unpaired bundle appeared (Fig. 5g, *arrows*).

In 10-segmented worms (Fig. 5h-﻿j, Additional file 3), 5-HT-lir structures in the anterior region were detected in the brain (*asterisk*), tentacular nerves (*open arrows*), subesophageal ganglion (*arrows*), ventral cord (*vc*) and parapodial nerves (*arrowheads*). At this time, 5-HT-lir innervation of the pharynx was detected (Fig. 5i). At the posterior body end, 5-HT-lir neurites were detected in the terminal commissure (*tc*) and innervated the anal cirri (Fig. 5j, *arrows*). No visible changes in the arrangement of 5-HT-lir nerve elements could be seen during the subsequent growth of the worms.

### Catecholamine histofluorescence (Figs. 6 and 7)

**Fig. 6:**
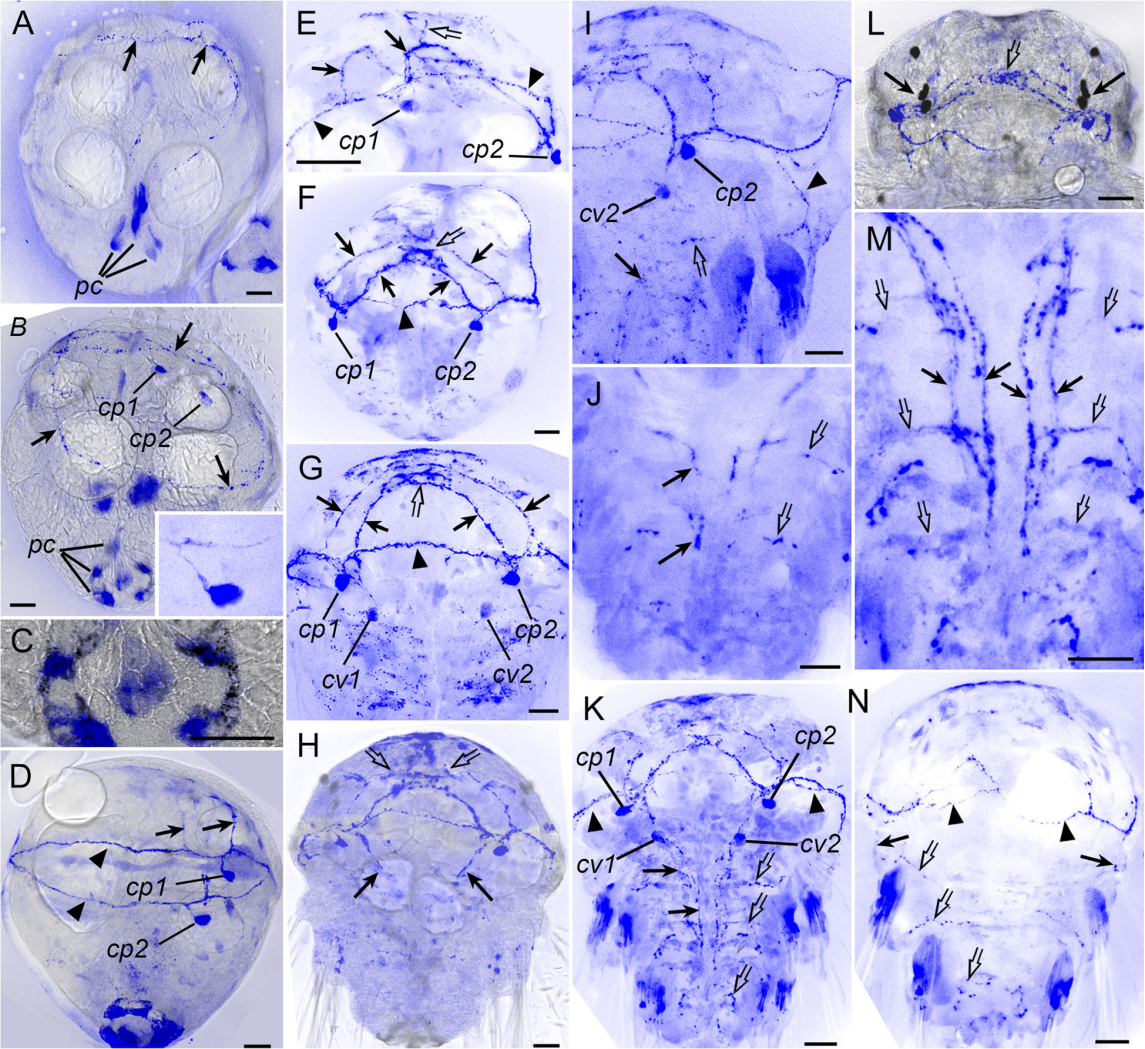
Development of catecholamine containing elements (*blue*) in *P. dumerilii*. Early trochophore (**a-c**), mid-trochophore (**d,e**), late trochophore (**f**), early metatrochophore (**g,h**), mid-metatrochophore (**i,j**), late metatrochophore (**k-n**). **a-d,h** and **l** are combinations of fluorescence (*blue*) and transmission light image (*grey*). **a:** 20 hpf, ventrolateral view showing CA-containing neurite under the prototroch (*arrows*) and six presumably non-nervous structures at the posterior body end (posterior cells, *pc*). **b:** 20 hpf, two CF-containing cells (*cp1,2*) and their fibers underlying the prototroch (*arrows*). Inset: high magnification of the cell *cp1*. **c:** 20 hpf, fluorescent structures at the surface of the posterior body end. **d:** 28 hpf, lateral view showing the cells *cp1,2* and CA-containing neurites in the circular prototroch nerve (*arrowheads*) and two brain commissures (*arrows*). **e:** 32 hpf, lateral view on the anterior hemisphere showing the cells *cp1,2* and their neurites in the circular prototroch nerve (*arrowheads*), two brain commissures (*arrows*) and a solitary fiber going towards the apical organ (*open arrowhead*). **f:** 36 hpf, ventral view showing the cells *cp1,2* and CA-containing fibers in the prototroch nerve, (*arrowheads*), two brain commissures (*arrows*) and developing brain (*open arrow*). **g:** 40 hpf, ventral view showing the cells *cp1,2* and *cv1,2*, and CA-containing fibers in the prototroch nerve, (*arrowhead*), two brain commissures (*arrows*) and developing brain (*open arrow*). **h:** 44 hpf, ventral view showing CA-containing neurites in the apical region (*open arrows*) and in the region of developing ventral cord (*arrows*). **i:** 48 hpf, ventral view on the left size of anterior body part showing the cells *cp2* and *cv2*, and CA-containing neurites in the developing ventral cord (*arrow*), right dorsolateral nerve (*arrowhead*), and transverse commissure in the first chaetigerous segment (*open arrow*). **j:** 48 hpf, ventral view on the posterior body part showing CA-containing neurites in the developing ventral cord (*arrows*) and segmental nerves in the second and third chaetigerous segments (*open arrows*). **k:** 56 hpf, ventral view showing the cells *cp1,2* and *cv1,2*, as well as CA-containing neurites in the prototroch nerve (*arrowheads*), ventral cord (*arrows*) and three segmental nerves (*open arrows*). **l:** 56 hpf, dorsal view on the apical part showing brain neuropil (*open arrow*) and neurites at the base of larval eyes (*arrows*). **m:** 56 hpf, ventral view on the posterior body part showing CA-containing neurites in the ventral cord (*arrows*) and three segmental nerves (*open arrows*). **n:** 56 hpf, dorsal view showing CA-containing neurites in the prototroch nerve (*arrowheads*), dorsolateral nerves (*arrows*) and segmental nerves (*open arrows*). Scale bars = 20 μm

In this method, the primary catecholamines, dopamine and noradrenaline, were converted to fluorescent 2-carboxymethyl-dihydroisoquinoline derivatives in a reaction with glyoxylic acid with subsequent air drying and heating. Blue-green fluorescence a characteristic for catecholamines, was first observed in a neurite under the prototroch at the time of hatching (Fig. 6a, *arrows*). Soon after hatching, two unipolar neurons were observed in the hyposphere close to the prototroch (catecholaminergic prototroch, *cp1,2*) with their neurites passing under the prototroch (Fig. 6b, *arrows*). Fluorescence was also observed in six cell-like structures in the posterior region of the trochophore (Fig. 6a–c, *pc*). No neurites were detected extending from these structures so we considered them to be non-nervous. The site of bright fluorescence was observed at the surface of the posterior end of the larva and was co-localized with the cilia of the telotroch (Fig. 6c). By the late trochophore stage, this fluorescence faded and became undetectable.

**Fig. 7:**
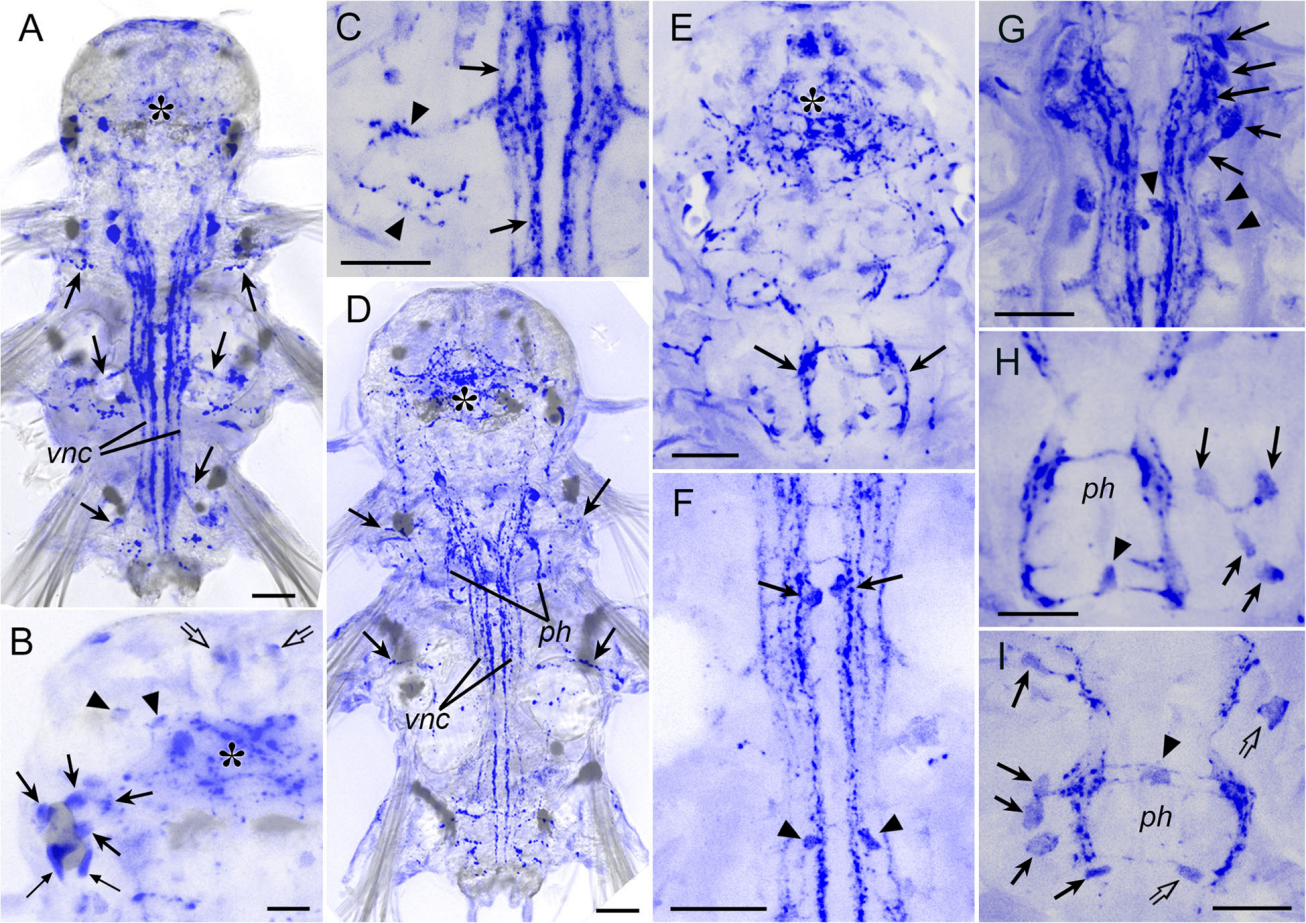
Development of catecholamine containing elements (*blue*) in *P. dumerilii*. Early nectochaete (**a-c**), mid-nectochaete (**d-f**), and late nectochaete (**g-i**); ventral views. **a, b** and **d** are combinations of fluorescence (*blue*) and transmission light image (*grey*). **a:** 72 hpf, CA-containing structures in the brain (*asterisk*), ventral nerve cord (*vnc*) and larval segments; *arrows* indicate segmental nerves. **b:** 72 hpf, the head region showing the brain neuropil (*asterisk*), two neurons in front of the brain (*open arrows*), two neurons to the right of it (*arrowheads*), and four neurons near the right eye (*arrows*). The pigmented eye looks grey and has two autofluorescent structures (*small arrows*). **c:** 72 hpf, high magnification showing CA-containing structures in the paired ventral cord (*arrows*) and parapodial nerves (*arrowheads*). **d:** 3.5 dpf, CA-containing structures in the brain (*asterisk*), pharynx (*ph*), ventral nerve cord (*vnc*), and parapodia (*arrows*). **e:** 3.5 dpf, dorsal part of the head showing CA-containing neurites in the brain (*asterisk*) and pharynx (*arrows*). **f:** 3.5 dpf, high magnification of the ventral cord showing two pairs of CA-containing cells in the first (*arrows*) and second (*arrowheads*) chaetigerous segments. **g:** 5 dpf, high magnification of the ventral cord showing five pairs of CA-containing cells (*arrows*) in the first chaetigerous segment and three pairs (*arrowheads*) in the second segment (only neurons in the left half of the cord are indicated). **h:** 5 dpf, high magnification of the dorsal part of the pharynx (*ph*) showing four CA-containing cells to the left of it (*arrows*), and one medial cell (*arrowhead*). **i:** 5 dpf, high magnification of the ventral part of of the pharynx (*ph*) showing five CA-containing cells to the right of it (*arrows*), two controlateral cells (open arrows) and one medial cell (*arrowhead*). Scale bars = 20 μm

At the mid-trochophore stage, fluorescent neurites gradually appeared in the first and second brain commissures (Fig. 6d,e, arrows). At the end of this stage, the first neurites extending towards the apical organ were detected (Fig. 6e, *open arrow*). In late trochophores (Fig. 6f), CA-containing neurites were detected in the prototroch nerve (*arrowhead*), two cerebral commissures (*arrows*), and the apical organ (*open arrow*).

At the early metatrochophore stage, two more cells (catecholaminergic ventral, *cv1,2*) were detected slightly posterior to the first pair in the region of the developing paired ventral cord (Fig. 6g). In the episphere, the number of CA-containing neurites at the base of the apical organ (cerebral neuropil), and in the two brain commissures gradually increased (Fig. 6g-h). At this stage, the first fluorescent neurites were detected in the hyposphere. Initially, they were scattered in the ventral region (Fig. 6g), and later they were seen to be the part of the paired ventral cord (Fig. 6h, *arrows*). In the mid-metatrochophores, CA-containing neurites in the hyposphere started to arrange into two dorsolateral nerves (*arrows*) and three transverse commissures (*open arrows*) corresponding to the rudiments of the chaetigerous segments (Fig. 6i-j).

At the late metatrochophore stage, no additional CA-containing cells were detected, though the number of fluorescent neurites gradually increased (Fig. 6k). The neurites formed a neuropil in the brain and at the base of the larval eyes (Fig. 6l, *arrows*). Two pairs of short fibres extended dorsally from the second brain commissure. Fluorescent neurites in each half of the ventral cord were combined into two bundles (Fig. 6m, *arrows*), and more fibres appeared in the transverse commissures (Fig. 6m, *open arrows*). At the dorsal side (Fig. 6n), fluorescent fibres were detected in the prototroch nerve (*arrowheads*), dorsolateral nerves (*arrows*) and transverse commissures (*open arrows*).

During the early nectochaete stage, the number of CA-containing cells and neurites in the adult CNS gradually increased (Fig. 7a). The brain neuropil grew in size, so the brain commissures could no longer be recognized. Fourteen CA-containing neurons appeared in the head (Fig. 7b): four neurons near each eye (*arrows*), two pairs on both sides of the brain neuropil (*arrowheads*), and a pair in front (*open arrow*). In the body (Fig. 7c), more CA-containing neurites appeared in the ventral cord (*arrows*) and parapodial nerves (*arrowheads*). Starting from the mid-nectochaete stage, more CA-containing structures appeared in the CNS and at the periphery (Fig. 7d). Besides the brain and ventral cord, fluorescent neurites were detected to innervate the anterior regions of the digestive tract (Fig. 7e, *arrows*). Fluorescent neurons began to appear in the ganglia of all three chaetigerous segments. First, one pair of neurons was detected in each (Fig. 7f), and later, their number increased, so at the late nectochaete stage (Fig. 7g), the first segment contained five pairs of neurons (*arrows*), the second had three pairs (*arrowheads*), and the third had two pairs. CA-containing cells were also detected in the anterior region of the digestive tract but significant deformation – which is inevitable during air drying – did not allow us to determine their number and precise location. Usually, four cells were visualised to the left or to the right of the dorsal part of the gut (Fig. 7h, *arrows*), and five cells to the left or to the right of the ventral part of the gut (Fig. 7i, *arrows*), although sometimes controlateral cells could be seen (Fig. 7i, *open arrows*). One medial cell was detected in the dorsal (Fig. 7h, *arrowhead*) and one in ventral part part of the gut (Fig. 7i, *arrowhead*).

### Dorsal sensory structure (Fig. 8)

**Fig. 8:**
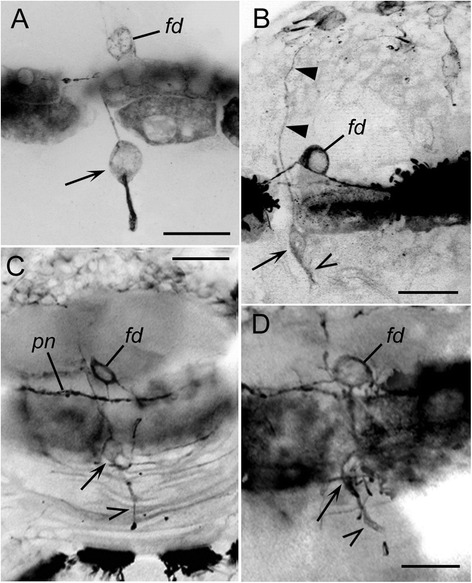
Development of the dorsal sensory structure in *P. dumerilii*. Mid-trochophore (**a**), late trochophore (**b**), early nectochaete (**c**), and mid-nectochaete (**d**). Dorsal views, acetylated tubulin-like immunoreactivities are presented as negatives. **a:** 28 hpf, bipolar neuron (*arrow*) and the cell *fd* are located dorsally on both sides of the prototroch and send fibres into the prototroch nerve. **b:** 36 hpf, the cell *fd* and the bipolar sensory cell (*arrow*) with the short apical fibre (*open arrowhead*) and basal fibre (*arrowheads*) running into the apical neuropil. **c:** 68 hpf, the cell *fd* and the bipolar sensory cell (*arrow*) with the apical ciliated fibre (*open arrowhead*); *pn* – prototroch nerve. **d:** 72 hpf, the above listed structures show signs of degeneration. Scale bars = 20 μm

Besides the apical sensory organ, another provisory sensory structure developed in the larval *P. dumerilii* (Fig. 8). At the mid-trochophore stage, anti-tubulin immunostaining revealed a bipolar cell of an unknown transmitter phenotype (Fig. 8a, *arrow*) close to the FMRFa-lir dorsal neuron *fd*. The soma of this cell was located slightly posterior to the prototroch nerve, a basal neurite ran into it, and a short apical fibre bore surface cilia. At the late trochophore stage, the bipolar sensory neuron sent a basal fibre into the apical neuropil (Fig. 8b, *double arrow*). This structure remained unchanged until the mid-nectochaete stage (Fig. 8c), and it later started to degenerate (Fig. 8d). Degeneration corresponded to the time when FMRFa-like immunoreactivity in the cell *fd* faded. At the late nectochaete stage, no trace of this structure was found.

## Discussion

Our study provides a detailed description of neuronal development in larval and juvenile polychaete *Platynereis dumerilii* including the ontogeny of serotonergic and catecholamine containing neuronal elements as well as FMRFa-like immunoreactive structures. Many members of the RFamide neuropeptide family are known to be present in a variety of invertebrate animals including *P.dumerilii* [51, 52]. The commercial anti-FMRFamide antibody that we used is known to target several epitopes shared by different members of the family and is not specific for particular FMRFamide related peptides [53]. Therefore it cannot be used to compare the distribution of specific peptides or track the evolution of the expression of certain neuropeptides genes. However, during the last few decades this antibody has been extensively used to study trochozoan neurogenesis (see refs above). We therefore consider these data useful for the comparative morphological analysis of neuronal development.

**Fig. 9:**
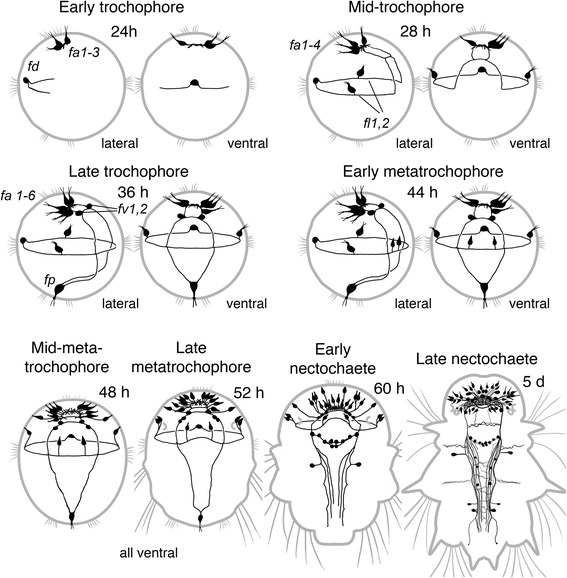
Summary diagram of the ontogeny of FMRFamide-like structures in *P. dumerilii*. The earliest FMRFa-lir somata appear in early trochophores. Basal neurites of the solitary dorsal neuron *fd* outline the future prototroch nerve. Three apical sensory neurons *fa 1–3* send basal fibers into apical neuropil. By the late trochophore stage, the set of larval sensory neurons is supplemented with a solitary posterior sensory cell *p1*, two lateral cells *fl 1–2*, and three more apical cells *fa 4–6*. Besides, the first pair of central neurons *fv 1,2* appears in the developing brain. Basal fibers of these cells outline the prototroch nerve, ventral cord and cerebral commissures. At the metatrochophore stage, many more sensory cells and additional central neurons appear in the episphere. Starting from the nectochaete stage, the adult CNS develops rapidly. Apical end is always up. Relative dimensions are not maintained

**Fig. 10:**
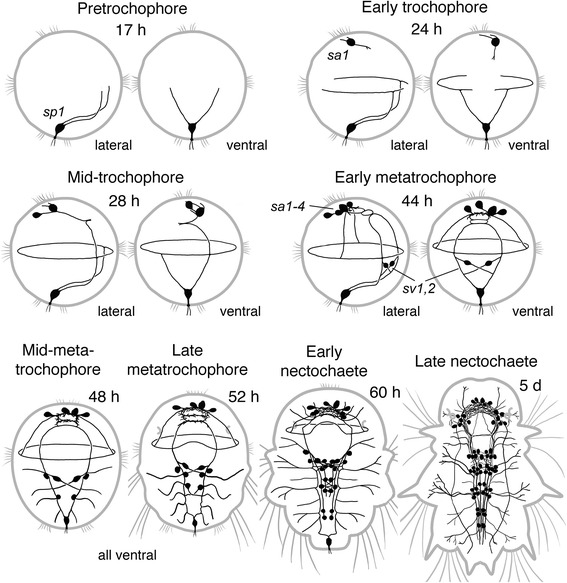
Summary diagram of the ontogeny of serotonergic structures in *P. dumerilii*. The earliest 5HT-lir posterior sensory cell *sp1* appears at the pretrochophore stage before hatching. Soon after hatching, a solitary sensory cell *sa1* appears at the apical extreme and basal fibers of these two cells outline the future prototroch nerve, paired ventral cord and brain commissure. In metatrochophores, more sensory cells add in the apical region and along the developing ventral cord. Starting from the nectochaete stage, 5HT-lir cells and neurites rapidly develop in the adult CNS. Apical end is always up. Relative dimensions are not maintained

**Fig. 11:**
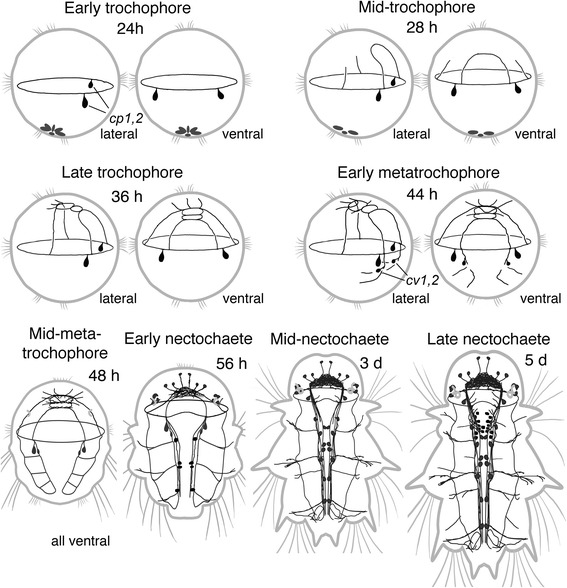
Summary diagram of the ontogeny of catecholamine containing structures in *P. dumerilii*. The first CA cells *cp1,2* appear in early trochophores slightly behind the prototroch. Their basal fibers run into the prototroch nerve, and by the late trochophore stage, they outline the prototroch nerve, pretrochal part of the ventral nerve cord and brain commissures. In metatrochophores, two more cells *cv1,2* appear and by the late metatrochophore stage, the fibers of these cells outline the brain and brain commissures, prototroch nerve, ventral cord and three transverse commissures corresponding to larval segments. Starting from the nectochaete stage, more cells and neurites add in the developing central and peripheral adult nervous system. Apical end is always up. Relative dimensions are not maintained

Our results on the development of serotonergic nervous elements are in accordance with the earlier study by Fischer and co-workers [11]. However, we present a more detailed description of 5-HT immunoreactive structures at the early stages of development. We also extended the study on the late developmental stages (juvenile worms). For all three chemically specific subsets of neurons we tried to identify the first neurons to appear as well as their projections and fate during metamorphosis (for the summary of neural development see Figs. 9, 10 and 11).

The first cell we considered to be a neuron appears shortly before hatching at the posterior extreme of the larva and expresses serotonin (*sp1*). No other neuron-like structures were detected at this time by anti-tubulin immunostaining thus suggesting this cell to be the first neuronal structure. The cell has a short apical neurite and two long basal fibers, which run towards the prototroch. After hatching, two cilia appear at the end of its short apical neurite indicating the sensory nature of this cell. At the late trochophore stage, a similar FMRFa-lir cell (*fp1*) appears and we suggest that *sp1* and *fp1* are the same cell, which is both 5-HT- and FMRFa-lir.

1. Its position, size and morphology (a short apical and two basal fibres) are similar after both 5HT-tubulin and FMRFa-tubulin immunolabeling.
2. The cell always bears cilia, and no other cilia were detected inside the telotroch ring (compare Fig. 1j and Fig. 4f). Thus, the first neuron probably expresses 5HT and an FMRFa related peptide, the latter transiently at the stages from late trochophore to late metatrochophore.

Soon after hatching, the cell *sp1* is supplemented with the apical cell *sa1*. The neurites of these cells pioneer pathways upon which the adult nervous system subsequently develops: the brain (cerebral ganglion), paired ventral nerve cord, and trochal neurite bundle (prototroch nerve) (see Fig. 4d). It has been suggested recently that in the course of Lophotrochozan larval development, the earliest neurons appear at the periphery and their basal neurites form the scaffold upon which the future adult nervous system later develops [54]. In *P.dumerilii*, both pioneer neurons are also peripheral cells and their basal neurites label the pathways where the adult CNS will later develop thus supporting the concept of pioneer neurons.

The 5-HT-lir neuron *sp1* is strikingly similar to those, found in other polychaete species *Phyllodoce maculata* [42], and *Pomatoceros lamarckii* [43]. In *Sabellaria alveolata*, the soma of the pioneer neuron was not visualized, but the neuronal processes were detected as two bundles along the ventral side and underneath the prototroch [44]. Thus, a solitary posterior serotonergic pioneer neuron is probably a common feature among polychaetes. However, no co-localization of 5-HT- and FMRFa-like immunoreactivity was found in *P. maculata*, and *P. lamarckii*. At the apical extreme, early peripheral FMRFa- and 5-HT-lir neurons were also found in almost all polychaete species studied to date [42–44, 55–58]. The only exception is *Owenia fusiformis*, which lacks 5 HT-lir cells in the apical organ [59, 60].

Two alternative hypotheses suggest that the annelid nervous system develops from either one anterior rudiment or two rudiments, anterior and posterior (for refs see [61]). Our data supports the idea that the scenario of larval neuronal development starting at two opposite poles, apical and caudal, could be a characteristic feature of polychaetes. An electron microscope study of early neuronal patterning in larval *Spirobranchus* has also revealed two initially separate parts of the nervous system: pretrochal and suboral [62]. The former precisely corresponds to early apical neurons in *P. dumerilii*, while the correspondence of the latter to the caudal system is not obvious. However, the description of *Spirobranchus* neuronal development by Lacalli started from later stages when caudal pioneering neurons could not be identified any more. Thus, we speculate that the suboral part of the nervous system in *Spirobranchus* is derived from the caudal rudiment. Earlier, the “chimeric brain hypothesis” suggested that insect, annelid, and vertebrate nervous systems evolved from two opposite aggregations of neurons (“apical nervous system” versus “blastoporal nervous system”) [39]. Our results are in agreement with this hypothesis.

The early dorsal FMRFa-lir cell *fd* has no analogues in other polychaete species studied to date. However, this cell is presumably the part of the dorsal sensory structure, which is very much like the posterior sensory organ found in another members of the order Phyllodocida, *P. maculata* [63]. Contrary to *P. dumerilii*, two 5-HT-lir and no FMRFa-lir neurons were found in the posterior sensory organ of *P. maculata*. The gross morphology, however, looks very similar when not counting the number of sensory neurons: one in *P. dumerilii* versus five in *P. maculata.* One can speculate that, as compared to phyllodocid larvae with a long planktotrophic stage, the reduced free-swimming period of lecitotrophic *Platynereis* larvae resulted in partial reduction of the provisory sensory structures.

The ventral nerve cords initially appear as paired fibres of the posterior cell *sp1* (Fig. 4 a-d), and the number of neurites in both cords gradually increases in the course of development. All histo- and immunostaining techniques exploited in this study revealed two symmetrical cords until the nectochaete stage (Figs. 1o,4h and 7f). Only in mid-nectochaetae, a central unpaired nerve bundle was detected after acetylated tubulin immunolabelling (Figs. 2a and 4i). However, paired symmetrical serotonergic nerve fibres were detected in the medial bundle even in 5- and 10-segmented juvenile worms (Fig. 5g,j). Thus, our data shows that the ventral nerve cord of *P. dumerilii* develops from initially paired nerves and the medial unpaired bundle appears later. It has been shown that during polychaete metamorphosis, the ventral neuroectoderm narrows and lengthens by mediolateral cell intercalation, and nerve bundles of the ventral cord cords approach each other [64]. Perhaps, for this reason, central bundles of the initially paired ventral cord come close and fuse together into the unpaired medial bundle. Thus, our results argue the suggestion that the unpaired median nerve belongs to the Annelida basic body plan [61, 65], and support the earlier idea of paired trunks within the ventral cord as the plesiomorphic condition [66].

CA-containing cells are sparse and, except the first pair *cp1, 2*, they appear at late developmental stages. No CA-containing cells were found in the apical organ at any stages. In general, the structure of all CA-containing neurons suggests they are not sensory: they are rounded and have no peripherally directed fibres. In other larval Trochozoa, CA-containing cells are usually sensory and located in the structures used for food detection: close to the mouth and on the internal surface of the ciliated lobes and tentacles [20, 23, 67–73]. We speculate that the lack of early sensory CA-containing cells in larval *P. dumerilii* resulted from transition to lecitotrophy.

The relatively small number of CA-containing cells in the ventral nerve cord is in accordance with the data for other polychaete species. Thus, only two and three pairs of such cells were found in *Nephtys* [45] and *Ophryotrocha puerilis* [46]. Similarly to *P. dumerilii*, four CA-containing nerve bundles were found in the ventral cord of *O. puerilis*, probably the functions of the catecholaminergic system in different polychaetes are similar.

In *P. dumerilii*, different parts of the future adult CNS are labelled by different pioneer neurons. Thus, according to our data, the paired ventral cord is outlined by the posterior serotonergic cell *sp1*, the prototroch nerve by the cells *sp1*, *fd* (FMRFa-lir dorsal), *fl1,2*(FMRFa-lir lateral) and *cp1,2* (CA-containing ventrolateral). The adult brain including the cerebral ganglion and brain commissures are labelled by the cells *fa1–4* (FMRFa-lir apical) and *sa1–4* (serotonergic apical). Early stages of neuronal development in other polychaete species are similar in that the fibres of the early pioneer neurons outline the main parts of the future adult CNS [42–44, 58]. However, in planctotrophic larvae, numerous additional transitory larval sensory cells and additional meridianal nerves appear [42, 74]. Despite a relatively long free-swimming period, *P. dumerilii* larvae are lecithotrophic. One can speculate that additional neurons in planktotrophic larvae are involved in feeding behaviour.

For a long time, the pygidium of annelids was considered to be a simple structure composed of epidermal cells only [75]. However, a much more complex organization of the pygidium was described recently in adult *P. dumerilii* [76]. Our data however, expands on the differences in larval development. Thus, the commissure that connects the terminal parts of the ventral nerve cords (see Fig. 5j) was first detected at the nectochaete stage (See Fig. 1s and 4i). The presence of the terminal commissure was earlier described in *Spirobranchus polycerus* [62]. We speculate that the terminal commissure is a part of the basic annelid body plan. However, further investigations on the development of the pygidium in different annelid species are necessary.

A recent study in the development of the nervous system in Solenogastres (Mollusca, Neomeniomorpha) has shown that it is very similar to that of *P. dumerilii*: first neurons appear at both anterior and posterior poles, and the neurites of these neurons run towards the prototroch and form the scaffold for the future ventral nerve chords, so the CNS differentiates from both anterior and posterior poles [24]. In Polyplacophorans, which are sometimes united with Solenogastres as a basal clade Aculifera [77, 78], the scaffold is formed by two pairs of lateral cells being both 5-HT- and FMRFa-lir, and the CNS differentiates in the anterior-to-posterior direction. At the trochophore stage, the nervous system consists of a large apical organ, which later degenerates, cerebral ganglion, and paired ventral and ventrolateral cords with transverse commissures. The cerebral ganglion and the cords are the parts of the adult nervous system. The prototroch nerve is absent at all stages [79].

The contour of the mussel nervous system is formed by the pair of FMRFa-lir neurons located at the apical extreme of the trochophore. The nervous system differentiates in the anterior-to-posterior direction. The nervous system of trochophores and veligers consists of the apical organ, cerebral ganglion connected with it, and a pair of ventral cords. The prototroch nerve is also absent [23].

In the opistobranch gastropod *Aplysia californica*, the adult CNS is outlined by four posterior FMRFa neurons, and the nervous system of the trochophore consists of apical 5-HT-lir neurons and two ventral cords only [22, 80, 81]. In the freshwater pulmonate gastropod *L. stagnalis*, the trochophore nervous system includes two apical neurons, three posterior neurons, and a pair of longitudinal cords [82]. In both species, the nervous system differentiates in the posterior-to-anterior direction.

Comparison of general patterns of neuronal development in annelids and molluscs suggests that, despite of some differences, the sequence of events in the differentiation and development of the nervous system are always as follows: early peripheral neurons differentiate and their fibres form a scaffold upon which the future adult nervous system will later develop. The larval nervous system then develops, and later the adult nervous system forms along the pathways outlined by the early neurons. Finally, the larval nervous system and the early neurons disappear or partially incorporate into the adult nervous system [16, 20, 22, 23, 42, 43, 79, 82, 83]. This similarity suggests that the last common ancestor of Annelida and Mollusca had a biphasic life cycle with a planktonic larva and benthic adult [40, 84].

According to the primary heteronomy concept, all larval chaetigerous segments in polychaetes form simultaneously while postlarval segments form in the anterior-to-posterior direction [85]. This concept is also supported by *Hox* and *ParaHox* gene expression patterns, which are different in larval and postlarval segments in *Alitta virens* [86–88]. Conversely, expression patterns of the genes *engrailed* and *wingless* show the same anterior-to-posterior expression pattern in both larval and postlarval segments [89]. Our data demonstrates that contrary to the primary heteronomy concept, neurons differentiate in the anterior-to-posterior direction not only in postlarval but also in larval segments of *P. dumerilii*. Earlier, similar scenario of commissure appearance in ventral ganglia of *Sabellaria alveolata* larva was demonstrated [44]. Thus, simultaneous appearance of larval chaetigerous segments and the pattern of *Hox* genes expression support the primary heteronomy concept, though the sequence of neuronal development and expression of several other genes involved in segment formation contradict it. More data on the functions of *Hox* genes in postlarval development is necessary to resolve this contradiction.

Recently, *P. dumerilii* became a popular model animal for neurobiological studies and several novel methods were developed for its study. Characterization of neuropeptidome (repertoire of conserved proneuropeptides), identification of neuropeptides, reconstruction of individual neurons and neuronal circuits using serial-section transmission electron microscopy, and high resolution whole-body registration of gene expression allowed identification of many chemically specific neuronal subsets involved in regulation of ciliary locomotion, spatial orientation, circadian rhythms and settlement of the larva [7, 38, 52, 90–96]. Molecular topography of these subsets suggests they are evolutionary conserved [38–40, 96]. Immunostaining with anti-serotonin and FMRFamide antibodies as well as catecholamine histochemical staining visualizes only a small fraction of the neurons in the developing nervous system and results in an incomplete picture of the nervous system. However, most of the data for other Annelida and Lophotrochozoa in general was obtained using these immune- and histoshemical staining techniques, so we considered the presented results important for the comparative analysis. Comparative studies of neuronal clusters in basal branching annelid groups with different types of development may be useful for better understanding of the evolution of the bilaterian brain and its developmental plasticity.

## Conclusions

We have presented a detailed description of *Platynereis dumerilii* neuronal development based on anti-acetylated tubulin, serotonin, and FMRFamide-like immunostaining as well as catecholamine histofluorescence. The results are summarized in Figs. 9, 10 and 11. Our data confirms and expands the existing results, and offers new information for comparative analysis of the nervous system ontogeny in Polychaeta and Trochozoa in general. According to our data, the development starts from the first pioneer neurons at the posterior and anterior poles of the larva, and the basal neurites of these neurons form a scaffold upon which the adult CNS later differentiates﻿, starting from both anterior and posterior poles. Larval chaetigerous segments form from the anterior-to-posterior direction, contradicting the primary heteronomy concept. Comparison with Mollusca reveals substantial similarity with early neuronal development in larval Solenogastres. More comparative data on larval neuronal development in different groups of Trochozoa are necessary. Being a highly morphologically diverse taxon, Annelida are of particular interest, especially the groups that belong to the so-called basal branching annelids such as Chaetopteridae, Magelonidae and Amphinomidae [97, 98].

## Methods

### *Platynereis dumerilii* culture

*P. dumerilii* larvae were obtained from the laboratory breeding culture as described by Fischer and Dorresteijn [99]. Artificial sea water (ASW, Red Sea Coral Pro Salt) was used to culture adults and embryos. After spawning, the embryos were cultivated in glass Petri dishes 150 mm in diameter. Larvae and juvenile worms were fed on hard-boiled and homogenized quail eggs, and homogenized spinach. The speed of *P. dumerilii* development is known to be highly temperature dependent, thus minor temperature fluctuations cause significant changes in the pace of development, and complicate the comparison between studies [10, 11]. To avoid this problem, we cultivated all animals and developing embryos in a climate chamber at 18 °C, so the schedule of development corresponded to that described by Fisher and co-authors [11].

### Sampling, fixation and staging

Samples were taken each hour slightly before and after hatching, every four hours until the nectochaete stage (ca. 3 days of development), and then every 12 h until the stage of small atokous worm (ca. 20–30 days). All larvae were collected on Nitex screens, rinsed in ASW, relaxed by gradually adding a 7.5% magnesium chloride aqueous solution, and fixed in fresh 4% paraformaldehyde in phosphate buffered saline (PBS) for 1 h at room temperature, washed in PBS, transferred into 70% ethanol and stored at −20°. Besides the timing of development, each stage was additionally determined by the set of morphological characters described in [11].

### Immunochemistry

After storage, the specimens were rinsed in PBS 3 × 15 min, blocked overnight in PBS with 10% normal goat serum, 0.25% bovine serum albumin, 1% Triton X-100 (TX), and 0.03% sodium azide, and incubated in a mixture of either anti-5-HT or anti-FMRFamide primary ABs (Immunostar, 20,080 and 20,091, respectively; both polyclonal and raised in rabbits and diluted 1:2000–3000) together with monoclonal anti-acetylated α-tubulin AB (Sigma, Cat. No. T-6793, developed in mouse, diluted 1:1000–1500) in a blocking solution for 1–3 days at 10 °C. The specimens were then washed in PBS and incubated in a mixture of goat anti-rabbit Alexa 488 conjugated IgG and goat anti-mouse Alexa 546 IgG (Molecular Probes) both diluted 1:800 in PBS-TX, overnight at 10 °C. The specimens were washed in PBS several more times and mounted on glass slides in glycerol or TDE [100]. If necessary, 2–5 μg/ml HOEHST 33258 (Invitrogen, H1398) was added to one of the last wash to label cell nuclei. This allowed unambiguous differentiation between neuron perikarya and aggregations of fibers. Replacement of the primary antibodies with non-immune serum did not result in any staining. Reversal of the colours of the secondary antibodies (anti-rabbit Alexa 546 IgG and anti-mouse Alexa 488 IgG) yielded identical staining patterns.

### Catecholamine histochemistry

A glyoxylic acid fluorescent technique [101] was employed to visualize catecholamine-containing cells. Embryos were immersed in a freshly prepared, buffered glyoxylic acid-sucrose solution (500 mM sodium glyoxylate, 150 mM sucrose, 50 mM Tris buffer, pH 7.4) on glass slides at 4 °C. After 60 min of incubation, the solution was removed, and the embryos were air-dried at room temperature for 30 min. Preparations were then heated to 60 °C for 30 min, embedded in paraffin oil, and examined using Leica TCS SP5 laser scanning microscope (excitation at 405 nm, emission detection at 457–490 nm). For illustrations, the images were converted into negatives, so fluorescence of catecholamines looks blue. Controls in which larvae were once more examined after addition of distilled water to the preparation, showed no fluorescence characteristic of catecholamines.

### Microscopy and image processing

All specimens were examined as whole-mounts on laser scanning microscopes Zeiss LSM 510, Leica TCS SP5 and Leica TCS SPE with high aperture oil immersion objectives using appropriate wavelength-filter configuration settings. No fewer than 100 embryos were examined at each stage for each of the antibodies. For each larva, 40–150 0.5 μm thick optical sections were taken and processed with Zeiss LSM IB, Leica LAS AF, Bitplane Imaris and ImageJ. Three-dimensional (3D), rotatable reconstructions were produced using Imaris and converted into AVI files. A series of optical sections were also projected into single images and exported as TIFF images. These images were then adjusted for contrast and brightness and assembled into plates using Adobe Photoshop CS.

## Additional files

Additional file 1: FMRFamide-like (green) and acetylated tubulin-like (magenta) immunoreactivity in the anterior body part of three-segmented juvenile of *P. dumerilii*, 3D rotatable reconstruction. (AVI 6162 kb)

Additional file 2: FMRFamide-like (green) and acetylated tubulin-like (magenta) immunoreactivity in the anterior body part of ten-segmented juvenile of *P. dumerilii*, 3D rotatable reconstruction. (AVI 4623 kb)

Additional file 3: 5-HT-like (green) and acetylated tubulin-like (magenta) immunoreactivity in the anterior body part of ten-segmented juvenile of *P. dumerilii*, 3D rotatable reconstruction. (AVI 3097 kb)

## References

[1] Aguinaldo AMA, Turbeville JM, Linford LS, Rivera MC, Garey JR, Raff RA (1997). Evidence for a clade of nematodes, arthropods and other moulting animals. Nature.

[2] Halanych K, Bacheller J, Aguinaldo A, Liva S, Hillis D, Lake J (1995). Evidence from 18S ribosomal DNA that the lophophorates are protostome animals. Science.

[3] Field KG, Olsen GJ, Lane DJ, Giovannoni SJ, Ghiselin MT, Raff EC (1988). Molecular phylogeny of the animal kingdom. Science.

[4] Kim CB, Moon SY, Gelder SR, Kim W (1996). Phylogenetic relationships of annelids, molluscs, and arthropods evidenced from molecules and morphology. J Mol Evol.

[5] Peterson KJ, Cameron RA, Davidson EH (2000). Bilaterian origins: significance of new experimental observations. Dev Biol.

[6] Tessmar-Raible K, Arendt D (2003). Emerging systems: between vertebrates and arthropods, the Lophotrochozoa. Curr Opin Genet Dev.

[7] Jékely G, Colombelli J, Hausen H, Guy K, Stelzer E, Nédélec F (2008). Mechanism of phototaxis in marine zooplankton. Nature.

[8] Hardege JD (1999). Nereidid polychaetes as model organisms for marine chemical ecology. Hydrobiologia.

[9] Dorresteijn AWC (1990). Quantitative analysis of cellular differentiation during early embryogenesis Of*platynereis dumerilii*. Rouxs Arch Dev Biol.

[10] Fischer A, Dorresteijn A (2004). The polychaete *Platynereis dumerilii* (Annelida): a laboratory animal with spiralian cleavage, lifelong segment proliferation and a mixed benthic/pelagic life cycle. BioEssays.

[11] Fischer AH, Henrich T, Arendt D (2010). The normal development of *Platynereis dumerilii* (Nereididae, Annelida). Front Zool.

[12] Dorresteijn AWC, O’Grady B, Fischer A, Porchet-Henneré E, Boilly-Marer Y (1993). Molecular specification of cell lines in the embryo of Platynereis (Annelida). Roux’s Arch Dev Biol.

[13] Fisher A, Dorresteijn AW, Hoeger U (1996). Metabolism of oocyte construction and the generation of histospecificity in the cleaving egg. Lessons from nereid annelids. Int J Dev Biol.

[14] Arendt D, Tessmar K, de Campos-Baptista M-IM, Dorresteijn A, Wittbrodt J (2002). Development of pigment-cup eyes in the polychaete *Platynereis dumerilii* and evolutionary conservation of larval eyes in Bilateria. Development.

[15] Ackermann C, Dorresteijn A, Fischer A (2005). Clonal domains in postlarval *Platynereis dumerilii* (Annelida: Polychaeta). J Morphol.

[16] Croll R. Insights into early molluscan neuronal development through studies of transmitter phenotypes in embryonic pond snails. Microsc Res Tech 2000;578:570–8. 10.1002/1097-0029(20000615)49:6<570::AID-JEMT7>3.0.CO;2-Q 10862113

[17] Wanninger A (2009). Shaping the things to come: ontogeny of lophotrochozoan neuromuscular systems and the tetraneuralia concept. Biol Bull.

[18] Jackson AR, MacRae TH, Croll RP (1995). Unusual distribution of tubulin isoforms in the snail *Lymnaea stagnalis*. Cell Tissue Res.

[19] Croll RP (2006). Development of embryonic and larval cells containing serotonin, catecholamines, and FMRFamide-related peptides in the gastropod mollusc *Phestilla sibogae*. Biol Bull..

[20] Dickinson AJG, Croll RP (2003). Development of the larval nervous system of the gastropod *Ilyanassa obsoleta*. J Comp Neurol.

[21] Nielsen C, Haszprunar G, Ruthensteiner B, Wanninger A (2007). Early development of the aplacophoran mollusc Chaetoderma. Acta Zool.

[22] Dickinson AJ, Croll RP, Voronezhskaya EE (2000). Development of embryonic cells containing serotonin, catecholamines, and FMRFamide-related peptides in *Aplysia californica*. Biol Bull.

[23] Voronezhskaya EE, Nezlin LP, Odintsova NA, Plummer JT, Croll RP (2008). Neuronal development in larval mussel *Mytilus trossulus* (Mollusca: Bivalvia). Zoomorphology.

[24] Redl E, Scherholz M, Todt C, Wollesen T, Wanninger A (2014). 2014. Development of the nervous system in Solenogastres (Mollusca) reveals putative ancestral spiralian features. EvoDevo.

[25] Temereva E, Wanninger A (2012). Development of the nervous system in *Phoronopsis harmeri* (Lophotrochozoa, Phoronida) reveals both deuterostome- and trochozoan-like features. BMC Evol Biol.

[26] Temereva EN, Tsitrin EB (2013). Development, organization, and remodeling of phoronid muscles from embryo to metamorphosis (Lophotrochozoa: Phoronida). BMC Dev Biol.

[27] Temereva EN, Tsitrin EB (2014). Organization and metamorphic remodeling of the nervous system in juveniles of *Phoronopsis harmeri* (Phoronida): insights into evolution of the bilaterian nervous system. Front Zool.

[28] Temereva EN, Tsitrin EB (2014). Development and organization of the larval nervous system in *Phoronopsis harmeri*: new insights into phoronid phylogeny. Front Zool.

[29] Gruhl A (2009). Serotonergic and FMRFamidergic nervous systems in gymnolaemate bryozoan larvae. Zoomorphology.

[30] Gruhl A (2010). Neuromuscular system of the larva of *Fredericella sultana* (Bryozoa: Phylactolaemata). Zool Anzeiger A J Comp Zool.

[31] Santagata S (2008). Evolutionary and structural diversification of the larval nervous system among marine bryozoans. Biol Bull.

[32] Altenburger A, Wanninger A (2010). Neuromuscular development in *Novocrania anomala*: evidence for the presence of serotonin and a spiralian-like apical organ in lecithotrophic brachiopod larvae. Evol Dev..

[33] Hessling R, Westheide W (2002). Are Echiura derived from a segmented ancestor? Immunohistochemical analysis of the nervous system in developmental stages of *Bonellia viridis*. J Morphol.

[34] Hessling R (2002). Metameric organisation of the nervous system in developmental stages of *Urechis caupo* (Echiura) and its phylogenetic implications. Zoomorphology.

[35] Wanninger A, Koop D, Bromham L, Noonan E, Degnan BM (2005). Nervous and muscle system development in *Phascolion strombus* (Sipuncula). Dev Genes Evol.

[36] Denes AS, Jékely G, Steinmetz PRH, Raible F, Snyman H, Prud’homme B (2007). Molecular architecture of annelid nerve cord supports common origin of nervous system centralization in bilateria. Cell.

[37] Arendt D, Denes AS, Jékely G, Tessmar-Raible K. The evolution of nervous system centralization Philos. Trans R Soc Lond B Biol Sci 2008;363:1523–1528. 10.1098/rstb.2007.2242 PMC2614231 18192182

[38] Tomer R, Denes AS, Tessmar-Raible K, Arendt D (2010). Profiling by image registration reveals common origin of annelid mushroom bodies and vertebrate pallium. Cell.

[39] Tosches MA, Arendt D (2013). The bilaterian forebrain: an evolutionary chimaera. Curr Opin Neurobiol.

[40] Marlow H, Tosches MA, Tomer R, Steinmetz PR, Lauri A, Larsson T (2014). Larval body patterning and apical organs are conserved in animal evolution. BMC Biol.

[41] Hay-Schmidt A (1995). The Larval Nervous System of *Polygordius lacteus* Scheinder, 1868 (Polygordiidae, Polychaeta): Immunocytochemical Data. Acta Zool.

[42] Voronezhskaya EE, Tsitrin EB, Nezlin LP (2003). Neuronal development in larval polychaete *Phyllodoce maculata* (Phyllodocidae). J Comp Neurol.

[43] McDougall C, Chen W-C, Shimeld SM, Ferrier DEK (2006). The development of the larval nervous system, musculature and ciliary bands of *Pomatoceros lamarckii* (Annelida): heterochrony in polychaetes. Front Zool..

[44] Brinkmann N, Wanninger A (2008). Larval neurogenesis in *Sabellaria alveolata* reveals plasticity in polychaete neural patterning. Evol Dev..

[45] Clark ME (1966). Histochemical Localization of Monoamines in the Nervous System of the Polychaete Nephtys. Proc R Soc B Biol Sci.

[46] Schlawny A, Hamann T, Müller MA, Pfannenstiel H-D (1991). The catecholaminergic system of an annelid (*Ophryotrocha puerilis*, Polychaeta). Cell Tissue Res.

[47] Schlawny A, Grünig C, Pfannenstiel H-D (1991). Sensory and secretory cells of *Ophryotrocha puerilis* (Polychaeta). Zoomorphology.

[48] Díaz-Miranda L, de Motta GE, García-Arrarás JE (1992). Monoamines and neuropeptides as transmitters in the sedentary polychaete *Sabellastarte magnifica*: actions on the longitudinal muscle of the body wall. J Exp Zool.

[49] Crisp KM, Klukas KA, Gilchrist LS, Nartey AJ, Mesce KA (2002). Distribution and development of dopamine- and octopamine-synthesizing neurons in the medicinal leech. J Comp Neurol.

[50] Richter S, Loesel R, Purschke G, Schmidt-Rhaesa A, Scholtz G, Stach T (2010). Invertebrate neurophylogeny: suggested terms and definitions for a neuroanatomical glossary. Front Zool.

[51] Santama N, Benjamin PR, Burke JF (1995). Alternative RNA splicing generates diversity of neuropeptide expression in thebrain of the snail Lymnaea: in situ analysis of mutually exclusive transcripts of the FMRFamide gene. Eur J Neurosci.

[52] Conzelmann M, Williams EA, Krug K, Franz-Wachtel M, Macek B, Jékely G (2013). The neuropeptide complement of the marine annelid *Platynereis dumerilii*. BMC Genomics.

[53] Voronezhskaya EE, Elekes K (2003). Expression of FMRFamide gene encoded peptides by identified neurons in embryos and juveniles of the pulmonate snail *Lymnaea stagnalis*. Cell Tissue Res.

[54] Voronezhskaya EE, Ivashkin EG (2010). Pioneer neurons: A basis or limiting factor of lophotrochozoa nervous system diversity?. Russ J Dev Biol.

[55] Brinkmann N, Wanninger A (2009). Neurogenesis suggests independent evolution of opercula in serpulid polychaetes. BMC Evol Biol.

[56] Meyer NP, Carrillo-Baltodano A, Moore RE, Seaver EC (2015). Nervous system development in lecithotrophic larval and juvenile stages of the annelid *Capitella teleta*. Front Zool..

[57] Helm C, Krause A, Bleidorn C (2015). Immunohistochemical investigations of the development of *Scoloplos armiger* (“intertidalis clade”) indicate a paedomorphic origin of *Proscoloplos cygnochaetus* (Annelida, Orbiniidae). Invertebr Biol.

[58] Helm C, Schemel S, Bleidorn C (2013). Temporal plasticity in annelid development–ontogeny of *Phyllodoce groenlandica* (Phyllodocidae, Annelida) reveals heterochronous patterns. J Exp Zool B Mol Dev Evol.

[59] Helm C, Vöcking O, Kourtesis I, Hausen H (2016). *Owenia fusiformis* – a basally branching annelid suitable for studying ancestral features of annelid neural development. BMC Evol Biol.

[60] Hay-Schmidt A (2000). The evolution of the serotonergic nervous system. Proc R Soc London Ser B Biol Sci.

[61] Orrhage L, Müller MCM (2005). Morphology of the nervous system of Polychaeta (Annelida). Hydrobiologia.

[62] Lacalli TC (1984). Structure and organization of the nervous system in the trochophore larva of Spirobranchus. Philos Trans R Soc Lond Ser B Biol Sci.

[63] Nezlin LP, Voronezhskaya EE (2003). Novel, posterior sensory organ in the trochophore larva of *Phyllodoce maculata* (Polychaeta). Proc Biol Sci.

[64] Steinmetz PRH, Zelada-Gonzáles F, Burgtorf C, Wittbrodt J, Arendt D (2007). Polychaete trunk neuroectoderm converges and extends by mediolateral cell intercalation. Proc Natl Acad Sci U S A.

[65] Müller MCM (2006). Polychaete nervous systems: Ground pattern and variations – cLS microscopy and the importance of novel characteristics in phylogenetic analysis. Integr Comp Biol.

[66] Bullock TH, Horridge GA (1965). Structure and function in the nervous systems of invertebrates.

[67] Shunkina KV, Zaytseva OV, Starunov VV, Ostrovsky AN (2015). Comparative morphology of the nervous system in three phylactolaemate bryozoans. Front Zool.

[68] Santagata S (2002). Structure and metamorphic remodeling of the larval nervous system and musculature of *Phoronis pallida* (Phoronida). Evol Dev.

[69] Croll RP, Jackson DL, Voronezhskaya EE (1997). Catecholamine-Containing Cells in Larval and Postlarval Bivalve Molluscs. Biol Bull.

[70] Croll RP, Voronezhskaya EE, Hiripi L, Elekes K (1999). Development of catecholaminergic neurons in the pond snail, *Lymnaea stagnalis*: II. Postembryonic development of central and peripheral cells. J Comp Neurol.

[71] Voronezhskaya EE, Hiripi L, Elekes K, Croll RP (1999). Development of catecholaminergic neurons in the pond snail, *Lymnaea stagnalis*: I. Embryonic development of dopamine-containing neurons and dopamine-dependent behaviors. J Comp Neurol.

[72] Hay-schmidt A (1990). Distribution of catecholamine-containing, serotonin-like and neuropeptide FMRFamide-like immunoreactive neurons and processes in the nervous system of the actinotroch larva of *Phoronis muelleri* (Phoronida). Cell Tissue Res.

[73] Hay-Schmidt A (1990). Catecholamine-containing, serotonin-like and neuropeptide FMRFamide-like immunoreactive cells and processes in the nervous system of the pilidium larva (Nemertini). Zoomorphology.

[74] Nezlin LP (2010). The golden age of comparative morphology: Laser scanning microscopy and neurogenesis in trochophore animals. Russ J Dev Biol.

[75] Anderson DT (1966). The comparative embryology of the Polychaeta. Acta Zool.

[76] Starunov VV, Dray N, Belikova EV, Kerner P, Vervoort M, Balavoine G (2015). A metameric origin for the annelid pygidium?. BMC Evol Biol.

[77] Smith SA, Wilson NG, Goetz FE, Feehery C, Andrade SCS, Rouse GW (2011). Resolving the evolutionary relationships of molluscs with phylogenomic tools. Nature.

[78] Kocot KM, Cannon JT, Todt C, Citarella MR, Kohn AB, Meyer A (2011). Phylogenomics reveals deep molluscan relationships. Nature.

[79] Voronezhskaya EE, Tyurin SA, Nezlin LP (2002). Neuronal development in larval chiton *Ischnochiton hakodadensis* (Mollusca: Polyplacophora). J Comp Neurol Wiley Online Library..

[80] Marois R, Carew TJ (1997). Ontogeny of serotonergic neurons in *Aplysia californica*. J Comp Neurol.

[81] Marois R, Carew TJ (1997). Fine structure of the apical ganglion and its serotonergic cells in the larva of *Aplysia californica*. Biol Bull.

[82] Croll RP, Voronezhskaya EE (1996). Early elements in gastropod neurogenesis. Dev Biol.

[83] AJG D, Nason J, Croll RP (1999). Histochemical localization of FMRFamide, serotonin and catecholamines in embryonic *Crepidula fornicata* (Gastropoda, Prosobranchia). Zoomorphology.

[84] Jägersten G (1972). Evolution of the Metazoan Life Cycle.

[85] Ivanoff PP (1928). Die Entwicklung der larvalsegmente bei den Anneliden. Z Morphol Okol Tiere.

[86] Kulakova M, Bakalenko N, Novikova E, Cook CE, Eliseeva E, Steinmetz PRH (2007). Hox gene expression in larval development of the polychaetes *Nereis virens* and *Platynereis dumerilii* (Annelida, Lophotrochozoa). Dev Genes Evol.

[87] Bakalenko NI, Novikova EL, Nesterenko AY, Kulakova MA. Hox gene expression during postlarval development of the polychaete *Alitta virens*. Evodevo 2013;4:13. 10.1186/2041-9139-4-13 PMC3734159 23635090

[88] Kulakova MA, Cook CE, Andreeva TF (2008). ParaHox gene expression in larval and postlarval development of the polychaete *Nereis virens* (Annelida, Lophotrochozoa). BMC Dev Biol.

[89] Prud’homme B, de Rosa R, Arendt D, Julien J-F, Pajaziti R, Dorresteijn AWC (2003). Arthropod-like expression patterns of engrailed and wingless in the annelid *Platynereis dumerilii* suggest a role in segment formation. Curr Biol.

[90] Conzelmann M, Jékely G (2012). Antibodies against conserved amidated neuropeptide epitopes enrich the comparative neurobiology toolbox. EvoDevo.

[91] Shahidi R, Williams EA, Conzelmann M, Asadulina A, Verasztó C, Jasek S (2015). A serial multiplex immunogold labeling method for identifying peptidergic neurons in connectomes. elife.

[92] Williams EA, Conzelmann M, Jékely G (2015). Myoinhibitory peptide regulates feeding in the marine annelid Platynereis. Front Zool.

[93] Conzelmann M, Offenburger SL, Asadulina A, Keller T, Munch TA, Jekely G (2011). Neuropeptides regulate swimming depth of Platynereis larvae. Proc Natl Acad Sci U S A.

[94] Randel N, Asadulina A, Bezares-Calderón LA, Verasztó C, Williams EA, Conzelmann M (2014). Neuronal connectome of a sensory-motor circuit for visual navigation. elife.

[95] Tosches MA, Bucher D, Vopalensky P, Arendt D (2014). Melatonin signaling controls circadian swimming behavior in marine zooplankton. Cell.

[96] Tessmar-Raible K, Raible F, Christodoulou F, Guy K, Rembold M, Hausen H (2007). Conserved sensory-neurosecretory cell types in annelid and fish forebrain: insights into hypothalamus evolution. Cell.

[97] Weigert A, Helm C, Meyer M, Nickel B, Arendt D, Hausdorf B (2014). Illuminating the base of the annelid tree using transcriptomics. Mol Biol Evol.

[98] Struck TH, Paul C, Hill N, Hartmann S, Hösel C, Kube M (2011). Phylogenomic analyses unravel annelid evolution. Nature.

[99] Fischer A, Dorresteijn A. Culturing *Platynereis dumerilii*. 2016. http://www.staff.uni-giessen.de/~gf1307/breeding.htm. Accessed 18 May 2017.

[100] Staudt T, Lang MC, Medda R, Engelhardt J, Hell SW (2007). 2,2′-Thiodiethanol: A new water soluble mounting medium for high resolution optical microscopy. Microsc Res Tech.

[101] Lindvall O, Björklund A (1974). The glyoxylic acid fluorescence histochemical method: a detailed account of the methodology for the visualization of central catecholamine neurons. Histochemistry.

